# Broadband fluorescence reveals mechanistic differences in excited-state proton transfer to protic and aprotic solvents†

**DOI:** 10.1039/d0sc03316b

**Published:** 2020-07-08

**Authors:** Pragya Verma, Arnulf Rosspeintner, Bogdan Dereka, Eric Vauthey, Tatu Kumpulainen

**Affiliations:** Department of Physical Chemistry, University of Geneva 30 Quai Ernest Ansermet Geneva Switzerland Tatu.Kumpulainen@unige.ch +41 22 379 65 18 +41 22 379 36 58

## Abstract

Excited-state proton transfer (ESPT) to solvent is often explained according to the two-step Eigen–Weller model including a contact ion pair (CIP*) as an intermediate, but general applicability of the model has not been thoroughly examined. Furthermore, examples of the spectral identification of CIP* are scarce. Here, we report on a detailed investigation of ESPT to protic (H_2_O, D_2_O, MeOH and EtOH) and aprotic (DMSO) solvents utilizing a broadband fluorescence technique with sub-200 fs time resolution. The time-resolved spectra are decomposed into contributions from the protonated and deprotonated species and a clear signature of CIP* is identified in DMSO and MeOH. Interestingly, the CIP* intermediate is not observable in aqueous environment although the dynamics in all solvents are multi-exponential. Global analysis based on the Eigen–Weller model is satisfactory in all solvents, but the marked mechanistic differences between aqueous and organic solvents cast doubt on the physical validity of the rate constants obtained.

## Abstract 2

Time-resolved broadband fluorescence facilitates direct observation of reaction intermediates in excited-state proton transfer to solvent in protic and aprotic solvents.

## Graphical abstract

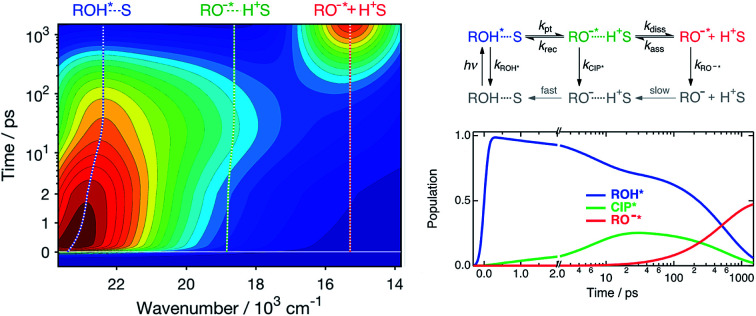

## 1 Introduction

Acid–base reactions, such as proton dissociation, transport and neutralization, play a pivotal role in several chemical, biological as well as technological processes.^1–9^ However, detailed investigation on the proton-transfer dynamics in solution is challenging due to the dynamic nature of the dissociation–association equilibrium. Excited-state proton transfer (ESPT) to solvent serves as a model system for the ground-state reaction and therefore remains as an active topic in chemical sciences.^10–15^

**Scheme 1:**
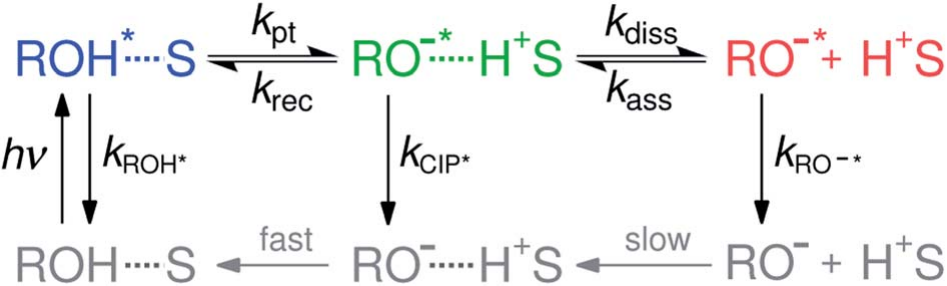
The Eigen–Weller model for excited-state proton transfer to solvent. The first step consists of a reversible short-range proton transfer from the protonated form (ROH*, blue) to solvent (S) producing contact ion pairs (CIP*, green) followed by a diffusion-controlled separation into free anions (RO^−^*, red). The ground-state species indicated in gray are not resolved in our study.

ESPT to solvent is often modeled according to the two-step Eigen–Weller model presented in Scheme 1.^16,17^ The first step consists of a short-range proton transfer from an excited photoacid (ROH*) to solvent producing contact ion pairs (CIP*). This is followed by the slower diffusion-controlled separation into free ions (RO^−^*). The model has been applied to explain ESPT to solvent in both aqueous and organic solvents.^18–22^ However, examples of a clear identification of the CIP* spectral signatures are scarce.^23–28^ Few studies reported an intermediate fluorescence between the ROH* and RO^−^* bands that was attributed to the contact ion pairs but this was observed only in supercritical,^23^ frozen or strongly acidic aqueous solutions^24,25^ and aprotic organic solvents.^26,27^ Therefore, it remains unclear whether the CIP* emission would be detectable in protic solvents at a moderate pH range. Identification of the CIP* emission in ultrafast ESPT reactions is further complicated by solvation dynamics. When the reaction rate approaches the time scale of the solvent dynamics, the reaction becomes solvent-controlled and is limited by the relaxation time of the solvent.^29–35^ This complicates the analysis because the single-wavelength fluorescence decays become contaminated by the spectral shifts due to solvent relaxation. Therefore, the individual contributions from the spectral shifts and population dynamics must be resolved separately for a detailed quantitative analysis.

**Fig. 1:**
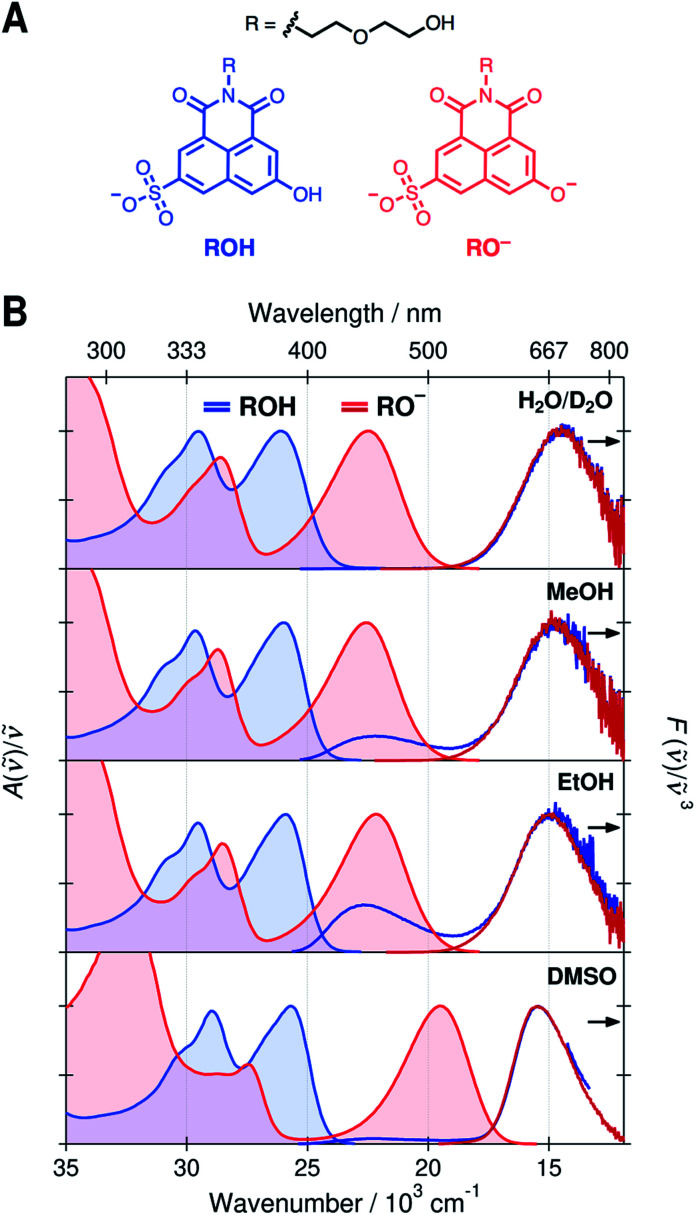
(A) Chemical structures of the protonated (ROH, blue) and deprotonated (RO^−^, red) forms of the photoacid, SHONI. (B) UV-vis absorption (solid lines with fill) and emission (dark solid lines) spectra of the ROH and RO^−^ forms of SHONI in D_2_O, MeOH, EtOH, and DMSO. All spectra are plotted in transition dipole moment representation. The excitation wavelengths were close to the S_1_ ← S_0_ absorption band maxima in all solvents.

Recently, we introduced a new global analysis approach for broadband fluorescence spectra and successfully applied it to model ESPT from a hydroxy-substituted 1,8-naphthalimide photoacid (SHONI, Fig. 1A) to DMSO.^35^ In the global analysis, each of the fluorescent species is modeled as a single time-dependent log-normal function.^22^ The main advantage of the global analysis is that the solvent relaxation can be modeled as frequency downshifts of the log-normal functions whereas the population dynamics can be independently accessed through the band integrals. Moreover, the band integrals can be coupled through rate equations derived from a reaction scheme, in this case the Eigen–Weller model, to achieve a so-called target analysis. Our study indicated that the initial formation of the contact ion pairs was largely driven by solvent relaxation resulting in excited-state equilibrium between the ROH* and CIP* forms. Secondly, the CIP* fluorescence was unambiguously identified in the time-resolved spectra.^35^

The photoacid, SHONI, exhibits an excited-state p*K*_a_ of −1.9 and is able to deprotonate in several organic solvents such as DMSO and alcohols.^36^ The choice of the solvent can impact ESPT in several ways. First, the proton accepting and solvating ability depends on the solvent therefore influencing the reaction free energy. The proton solvation energy is significantly lower in alcohols than in water or DMSO.^37^ Secondly, most solvents have vastly different solvent relaxation times ranging from *ca.* 1 ps in water up to few tens of ps in alcohols.^31,38^ Therefore, studies in a range of solvents provide significant insight into the influence of solvent relaxation on the ESPT reaction as has been demonstrated for other strong photoacids.^31,32^

The main goal of our study is to investigate the impact of protic (H_2_O, D_2_O, MeOH and EtOH) and aprotic (DMSO) solvents on the dynamics and mechanism of ESPT to solvent with a particular focus on testing the general applicability of the Eigen–Weller model. This is achieved by kinetic modeling of the broadband fluorescence spectra using the recently introduced global target analysis. Secondly, we present an attempt for resolving the CIP* fluorescence by decomposing the overall spectra into contributions from the protonated and deprotonated species. Last, we compare the results of the global target analysis to the experimental decay kinetics. Combination of these approaches provides an unprecedented mechanistic insight into ESPT to different solvents and allows us to rationalize the results of the global analysis and identify several limitations of the Eigen–Weller model.

## 2 Results and discussion

### 2.1 Steady-state spectra and transition dipole moments

Normalized absorption and emission spectra of the protonated (ROH) and deprotonated (RO^−^) forms of SHONI are presented in Fig. 1. The spectra are plotted in the transition dipole moment (TDM)^39^ representation similarly to the time-resolved fluorescence spectra and all spectral parameters (Table S1, ESI†) refer to these representations. The spectra in H_2_O and D_2_O are nearly indistinguishable and only those in D_2_O are shown for clarity.

The absorption spectra show two main bands attributed to the S_1_ ← S_0_ and S_2_ ← S_0_ transitions.^35,36^ In the RO^−^ form, both bands are shifted to a lower frequency and the energy gap between the bands increases. The shapes and frequencies of all spectra are relatively similar with the exception of the RO^−^ absorption in DMSO. Both bands exhibit a substantial red shift compared to protic solvents. This has been attributed to the absence of a direct hydrogen bond (HB) between the deprotonated hydroxyl oxygen and the solvent resulting in large destabilization of the electronic ground state.^35^ Due to this destabilization, the fluorescence spectrum is expected to undergo a similar red shift of *ca.* 3000 cm^−1^ upon cleavage of the direct hydrogen bond. In the Eigen–Weller model, the HB cleavage can be associated with a transition from the contact ion pairs to the fully separated ions.

The emission spectra in all solvents exhibit two bands at around 22 × 10^3^ cm^−1^ and 15 × 10^3^ cm^−1^ attributed to the protonated and deprotonated forms, respectively. The bands are slightly narrower and blue shifted in DMSO compared to protic solvents. The broadening of the spectra in protic solvents can be attributed to hydrogen-bonding interactions. The main difference in the emission spectra is however the ratio between the ROH* and RO^−^* emission intensities upon excitation of the ROH* form. The relative intensity of the high-frequency ROH* band increases in alcohols and is highest in EtOH whereas it is barely detectable in H_2_O, D_2_O and DMSO. This already suggests that the ESPT is significantly decelerated in alcohols. It should be noted, however, that the relative band intensities are not directly proportional to the populations but depend additionally on the relative fluorescence quantum yields of the species.

On the contrary, the time-resolved fluorescence band integrals in the TDM representation are directly proportional to the populations of the species scaled by their squared transition dipole moments (|*M*|^2^).^33^ If the TDMs are known, the relative concentrations can be obtained from the time-resolved spectra. In our previous study, we suggested that in DMSO the relative TDMs of the anionic species, CIP* and RO^−^*, were slightly smaller compared to that of ROH* form.^35,40^ We have now determined the TDMs from the steady-state spectra for both the ROH and RO^−^ forms. The values are identical within the uncertainty (<10%) regardless of the protonation state in all solvents with a mean value of |*M*| ≈ 2.8 D (Table S2, see ESI for details†).^41^ Therefore, the band integrals are directly proportional to the relative concentrations without additional scaling factors facilitating a full target analysis of the reaction dynamics.

### 2.2 Time-resolved fluorescence

**Fig. 2:**
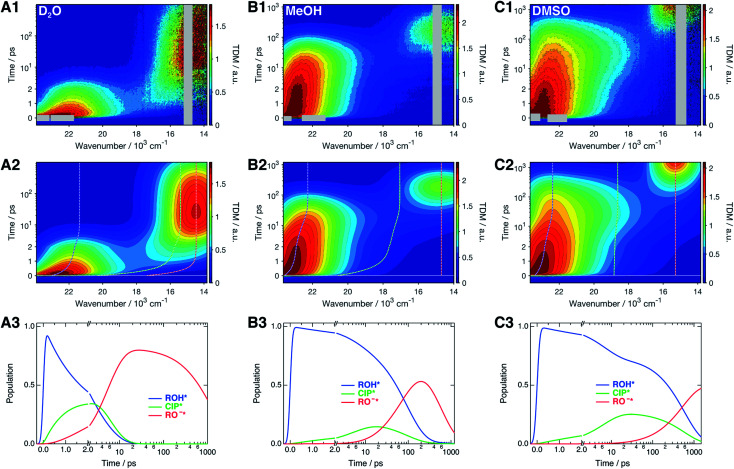
(1) Representative time-resolved fluorescence spectra, (2) global fits, and (3) concentration profiles of the excited-state species of SHONI in slightly acidified (A) D_2_O, (B) MeOH and (C) DMSO. The fits and concentration profiles were obtained from the global target analysis according to Scheme 1 with the resulting rate constants given in Table 1. Details of the analysis are given in ESI.† The gray areas are excluded from the fit due to pump scattering, Raman scattering and third harmonic of the gate pulse. The dashed lines in the middle panels indicate the time evolution of the band maxima of each species. Excitation was at 400 nm in all solvents.

The time-resolved fluorescence spectra of SHONI were measured in slightly acidified D_2_O, MeOH and DMSO and are presented in the top panels of Fig. 2. Supplementary data including both 2D- and 3D-plots of the spectra, fits and residuals in all solvents are presented in ESI.† The spectra in all solvents exhibit the prompt ROH* emission at around 22–23 × 10^3^ cm^−1^. During the first few ps in H_2_O and D_2_O and few tens of ps in other solvents, the ROH* band undergoes a red shift and decreases in intensity. In H_2_O and D_2_O, the ultrafast decay is accompanied by an equally fast appearance of the RO^−^* emission below 18 × 10^3^ cm^−1^. The decay is significantly faster in H_2_O resulting in a much higher intensity at around 18 × 10^3^ cm^−1^ during the first few ps. In alcohols and DMSO, the initial ROH* decay results in broadening of the ROH* band, which is particularly noticeable in MeOH and DMSO at around 18–20 × 10^3^ cm^−1^, whereas the majority of the RO^−^* emission appears on a longer time scale. Finally, the red shifted RO^−^* emission is observed as the last decaying species in all solvents.

### 2.3 Global target analysis based on the Eigen–Weller model

The spectra were analyzed globally using the target model presented in Scheme 1 (see ESI for details†). Based on the overall fit quality, the diffusion-controlled second step was assumed to be reversible in MeOH and EtOH whereas inclusion of *k*_ass_ did not significantly improve the fit in other solvents. The solvent relaxation was assumed to follow a bi-exponential function in all solvents and the mean solvation times of the ROH* form are shown in Table 2. In H_2_O and D_2_O, ESPT is extremely fast and proceeds on the same time scale as the solvent relaxation. As a result, the ROH* population decreases substantially during the first few ps compromising a detailed analysis of the solvation dynamics. Therefore, the time constants of the dynamic Stokes shift in H_2_O and D_2_O were determined independently upon direct excitation of the RO^−^* form (Fig. S2, ESI†) and used as constants in the analysis of the ESPT dynamics. The best global fits are presented in the middle panels of Fig. 2 and the resulting concentration profiles in the bottom panels. The overall agreement between the global fit and experimental spectra is very good in H_2_O and D_2_O but satisfactory in organic solvents. The dashed lines in the middle panels indicate the time evolution of the band positions of each species. The rate constants resulting from the global analyses are summarized in Table 1 and the band shape parameters of all species in Table S2 (ESI†).

**Table 1:** Summary of the kinetic parameters obtained from the global target analysis of the broadband fluorescence data^a^

Solvent	(*k*_pt_)^−1^/ps	(*k*_rec_)^−1^/ps	(*k*_diss_)^−1^/ps	(*k*_ass_)^−1^/ns	(*k*_ROH*_)^−1^^b^/ps	(*k*_CIP*_)^−1^^c^/ns	(*k*_RO^−^*_)^−1^/ns	*χ*_r_^2^
H_2_O	1.6 ± 0.2	5.6 ± 0.7	2.9 ± 0.3	—	4.3 ± 0.3	0.45 ± 0.02	0.45 ± 0.02	2.00
D_2_O	2.4 ± 0.2	5.7 ± 0.7	3.2 ± 0.3	—	15 ± 3	1.3 ± 0.2	1.3 ± 0.2	2.08
MeOH	35 ± 6	11 ± 2	21 ± 4	2.2 ± 0.3^d^	480 ± 170	0.5 ± 0.1	0.5 ± 0.1	2.39
EtOH	100 ± 10	40 ± 10	68 ± 6	1.2 ± 0.1^d^	800 ± 230	1.0 ± 0.1	1.0 ± 0.1	2.16
DMSO	25 ± 3	9 ± 2	280 ± 30	—	1000 ± 100	15.4^e^	15.4^e^	3.08

a The uncertainties represent the 95% confidence intervals obtained from the weighted fits.

b The direct decay of ROH*, *k*_ROH*_, reflects the total decay rate including any additional quenching processes. The rate constant without quenching processes in MeCN is (*k*_ROH*_)^−1^ = 3000 ps.

c The direct decay of CIP* was set equal to the decay of RO^−^* (*k*_CIP*_ = *k*_RO^−^*_).

d Reversibility of the second step was included in the model.

e The rate constant was constrained to the value determined from the fluorescence lifetime; see ESI for full details on the fitting procedures and boundary conditions used.

**Table 2:** Relative amplitudes and decay times of the protonated (ROH*) form after compensating for the spectral shift. The mean solvation times of the ROH* form are shown for comparison

Solvent	*α*_1_	*τ*_1_/ps	*α*_2_	*τ*_2_/ps	*α*_3_	*τ*_3_/ps	〈*τ*_solv_〉^a^/ps
H_2_O	0.60	0.7	0.40	2.5	—	—	0.8
D_2_O	0.37	0.8	0.63	3.9	—	—	1.1
MeOH	0.09	5.1	0.42	55	0.49	130	6.7
EtOH	0.20	29	0.75	220	0.05	1500	25
DMSO	0.21	3.9	0.10	36	0.69	600	5.2

a The value corresponds to the mean solvation time of the ROH* form calculated from the best-fit parameters of the global analysis according to 〈*τ*_solv_〉 = Σ*α*_*i*_*τ*_*i*_.

ESPT proceeds at a much higher rate in aqueous environment than in alcohols and DMSO. In H_2_O and D_2_O, the forward rate (*k*_pt_) is significantly larger than the backward rate (*k*_rec_) indicating a negative standard Δ*G*° for the forward reaction. The kinetic isotope effect is mostly observed in *k*_pt_ that is 1.5 times larger in H_2_O. In alcohols and DMSO, the reverse rate *k*_rec_ is 2 to 3 times higher than *k*_pt_ and the equilibrium is shifted towards the ROH* form. This shows that the standard reaction free energy is positive in organic solvents. In alcohols, the rate constant of the diffusion-controlled separation (*k*_diss_) is also higher than *k*_pt_. This indicates that the formation of free ions is largely limited by the relatively low transient concentration of the CIP* form. In addition, the reversibility of the second step further decelerates the formation of free ions although *k*_ass_ is nearly two orders of magnitude smaller than the forward rate. On the other hand, the rates of the initial deprotonation, *k*_pt_ and *k*_rec_, are significantly larger in DMSO compared to alcohols whereas the diffusion-controlled separation is much slower without any indications of reversibility.

Significant differences are also observed in the direct decay rates, *k*_ROH*_ and *k*_RO^−^*_. In the fit, the decay of CIP* was assumed to be equal to that of RO^−^* (*i.e. k*_CIP*_ = *k*_RO^−^*_). Therefore, any additional quenching processes will be included in the decay of the ROH* form.^42^ The decay rates of both the ROH* and RO^−^* forms increase in protic solvents being highest in H_2_O. The kinetic isotope effect on both decay rates is *k*_H_2_O_/*k*_D_2_O_ ≈ 3. Surprisingly, the decay of the RO^−^* form does not coincide with the fluorescence lifetime measured in basic MeOH and EtOH (Table S2, ESI†). This might partly result from the strict constraint on *k*_CIP*_. Secondly, the time-resolved experiments were performed on slightly acidified solutions of concentrated SHONI. The sulfonic acid group of SHONI might additionally lower the pH of the solutions.

We have previously observed that the fluorescence of SHONI is quenched by protons but additionally the proticity of the solvent appears to play a significant role.^36^ Actually, fluorescence quenching by protic solvents has been reported for several organic dyes but the exact mechanism has not been fully elucidated.^43–52^ One common factor in all cases is the presence of strong hydrogen bonds between the solute and the protic solvent. Moreover, the HB strength in most cases increases significantly in the excited state as a result of intramolecular charge-transfer (CT) type excitation. In such systems, dissipation of the excitation energy to the solvent as heat has been directly observed.^51^

The S_1_ state of SHONI has a significant CT-character with increased electron density on the carbonyl oxygens.^35,36^ Therefore, strong hydrogen bonding to the carbonyl groups is a likely cause for the increased decay rate of the RO^−^* form. However, the much larger decay rate of the ROH* form suggests that strong HB interactions with the hydroxyl group additionally contribute to the quenching process. Indications of such strong hydrogen bonds were observed in the time-resolved infrared absorption spectra as broad continuum signals in D_2_O, MeOH and DMSO (see ESI for details†). A similar continuum signal has been observed for a pyrenol-based photoacid, HPTS, in H_2_O and D_2_O where it was attributed to the hydroxyl proton that is partially shared with a water molecule due to the strong hydrogen bond.^53,54^ Therefore, it is likely that the deactivation indeed proceeds through energy dissipation to the solvent in the strongly hydrogen-bonded complexes.

### 2.4 Spectral identification of the CIP* intermediate

**Fig. 3:**
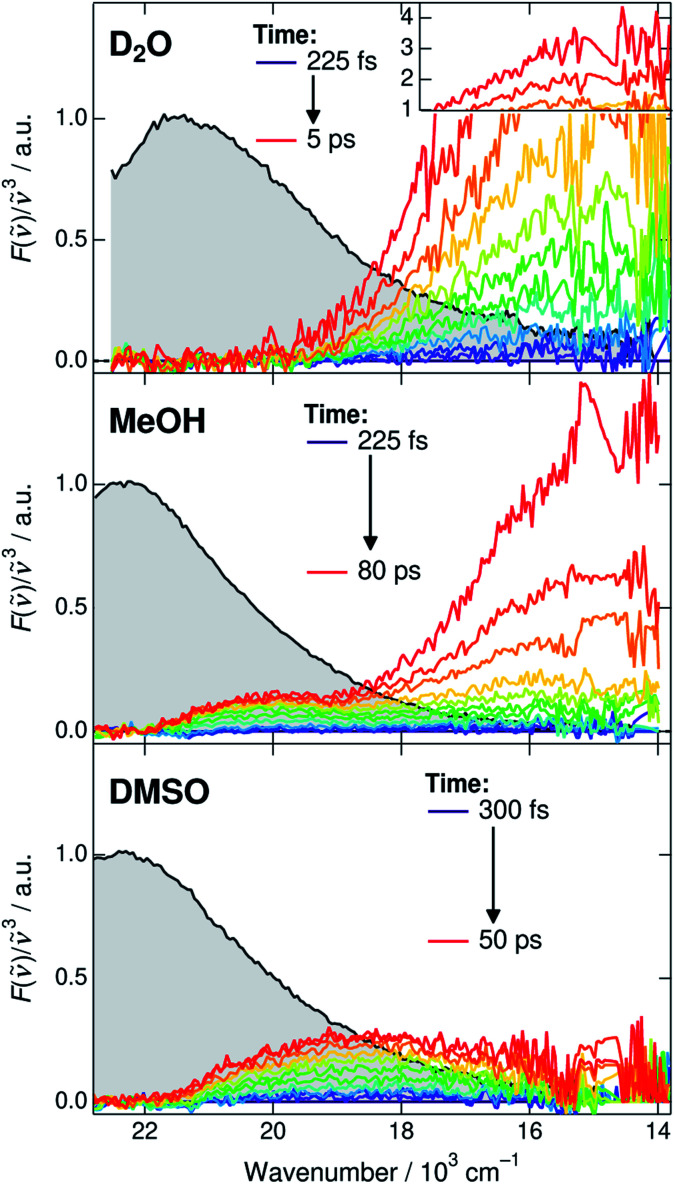
Time-resolved fluorescence spectra of the deprotonated species (CIP* and RO^−^*). The gray shaded areas indicate the fluorescence of the ROH* form at 225 or 300 fs that are subtracted from the subsequent spectra. The high intensity spectra in D_2_O have been scaled above 1. Details of the subtraction procedure and ESI spectra are given in ESI.†

Before discussing the time-dependent spectral properties, we first present our approach for extracting the fluorescence spectra of the deprotonated species (CIP* and RO^−^*). This is achieved by subtracting the ROH* fluorescence after accounting for the dynamic Stokes shift due to solvent relaxation.^35^ The time-resolved spectra were first shifted to have the same fluorescence peak maximum of the ROH* form at each time step. The spectra were subsequently normalized at the ROH* band maximum and the first spectrum was subtracted from the following spectra. The subtracted spectra in D_2_O, MeOH and DMSO are presented in Fig. 3 and represent the relative intensity with respect to the normalized ROH* fluorescence indicated in gray. The full procedure and supplementary spectra in all solvents are given in ESI.†

The extracted spectra reveal rather surprising and insightful differences in the CIP* and RO^−^* fluorescence in different solvents. In aqueous environment, no distinct CIP* band is observed. The long-wavelength fluorescence appears directly below 19 × 10^3^ cm^−1^ without significant shifts. According to the global target analysis, the CIP* population increases up to 40% but only a single deprotonated species is spectrally distinguishable. This indicates that the CIP* intermediate, if present, is extremely short-lived and hence thermodynamically unfavorable. Secondly, the Grotthuss-type proton-hopping mechanism can facilitate a direct escape of the proton from the ROH* form bypassing the CIP* intermediate.^55^

On the contrary, the spectra in DMSO show the appearance of a distinct CIP* fluorescence band that is well separated from the RO^−^* band (not visible in Fig. 3). Due to relatively small *k*_diss_ in DMSO, the RO^−^* band appears on a much longer time scale. In MeOH and EtOH, the spectra show both the appearance of the intermediate CIP* band as well as a fast rise of the red shifted RO^−^* band. This could be partly explained by a fast diffusion-controlled separation *k*_diss_ compared to *k*_pt_ but it also seems that the direct deprotonation *via* the proton-hopping mechanism is operative in alcohols. The CIP* band maximum provides additional information about the structure of the CIP* intermediate. In MeOH, the band maximum is closer to the ROH* form (∼20.0 × 10^3^ cm^−1^) whereas it is shifted more towards the RO^−^* form in DMSO (∼18.5 × 10^3^ cm^−1^). Such a shift results from a stronger stabilization of the excited-state product (and destabilization of the ground state) making the reaction thermodynamically more favorable. This is usually accompanied by increased hydrogen-bond length between the deprotonated photoacid and the protonated acceptor.^27,56^

The above observations allow us to rationalize the spectral shifts obtained from the global analysis (Fig. 2, middle panels). In aqueous solutions, the resulting CIP* band exhibits the largest dynamic Stokes shift and merges with the RO^−^* band. In DMSO, the global analysis correctly reproduces the intermediate CIP* and final RO^−^* bands centered at around 18–19 × 10^3^ cm^−1^ and 15 × 10^3^ cm^−1^, respectively. In MeOH and EtOH, the global analysis cannot correctly model both the intermediate CIP* band as well as the direct deprotonation. Therefore, the resulting CIP* band partly accounts for the direct deprotonation pathway and the relaxed band frequency is red shifted to 17 × 10^3^ cm^−1^ towards the RO^−^* band maximum. Due to this discrepancy, the resulting rate constants are less reliable and probably represent an average behavior between the two competing pathways. We tested a branched kinetic model to account for the complex dynamics but no meaningful fits were achieved. Moreover, it is likely that the solvation and reaction dynamics are not perfectly separated due to inadequate modeling of the reaction dynamics. In EtOH, the situation is even worse due to the much weaker and less distinct CIP* fluorescence and the modeled CIP* band largely accounts for the direct deprotonation pathway.

### 2.5 Comparison with experimental decays

**Fig. 4:**
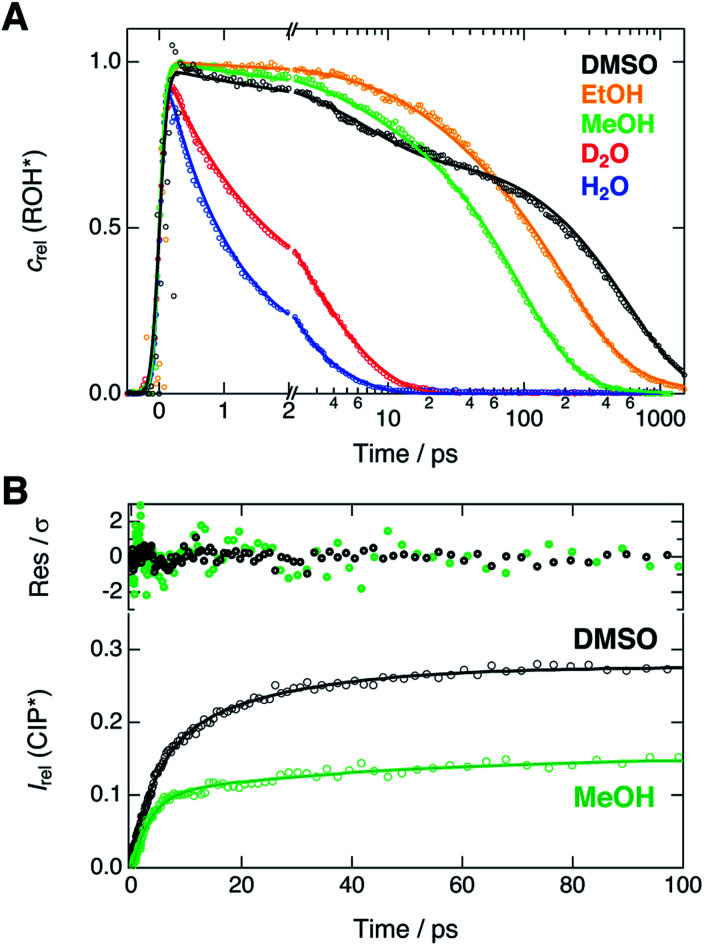
(A) Experimental decays (markers) of the ROH* fluorescence after compensating for the spectral shifts together with the corresponding relative concentrations (lines) obtained from the global analysis. (B) Rise of the relative CIP* fluorescence (markers) obtained from the extracted spectra presented in Fig. 3. The lines represent the best bi-exponential fits with time constants given in the text. The top panel shows the weighted residuals.

Next, we compare the experimental decays with the results of the global analysis. We extracted the ROH* population decays around the ROH* band maximum from the shifted spectra (*vide supra*). Therefore, the decay traces are not contaminated by the spectral shifts and represent the true population dynamics. Relative concentrations of the ROH* form resulting from the global analysis are overlaid with the experimental decays in Fig. 4A.

The decays were additionally analyzed with multi-exponential functions in order to extract the time scale of the ROH* decay. In the Eigen–Weller model, the ROH* population is expected to follow a bi-exponential decay where the first time constant is associated with the formation of contact ion pairs and the second with the diffusion-controlled separation into free ions.^20,35^ Therefore, the CIP* rise kinetics should coincide with the initial ROH* decay. Experimental CIP* rise in DMSO and MeOH was extracted from the subtracted spectra (Fig. 3) around the band maximum and are presented in Fig. 4B. The exponential analysis additionally allows us to discuss the influence of the solvent relaxation on the reaction. The exponential decay parameters and mean solvation times of the ROH* form are summarized in Table 2. The experimental decay traces of the ROH* and the RO^−^* forms together with the multi-exponential fits are additionally given in ESI.†

In H_2_O and D_2_O, the ROH* population decay is largely bi-exponential and the agreement between the experimental decays and relative concentrations is excellent. The time constant of the fast decay component is slightly shorter than the mean solvation time in both solvents but its amplitude is significantly smaller in D_2_O. The time constants demonstrate that the initial ESPT is controlled by the solvent relaxation and the slightly higher rate in H_2_O can be attributed to faster solvation dynamics. However, since the dielectric properties of the two solvents are nearly identical, the isotope effect on the amplitudes likely originates from differences in the zero-point energies.^57^ The deuterium isotope effect due to zero-point energies has been determined both experimentally and computationally to be around 0.5 p*K*_a_ units for several aromatic acids with varying acidities.^58^ This corresponds to a difference of 0.67 kcal mol^−1^ in Δ*G*. If the observed amplitudes of the fast decay components in H_2_O and D_2_O are assumed to reflect the relative CIP* populations, the difference in Δ*G* is estimated to be 0.55 kcal mol^−1^, in close agreement with the above value.

The slower decay time in H_2_O, on the other hand, coincides with the time scale of a global hydrogen-bond rearrangement or proton hopping events observed in several time-resolved studies.^59–61^ This shows that the slower deprotonation pathway involves a significant reorientation of the surrounding water molecules beyond the first solvation shell. Secondly, the deprotonation occurs *via* the hopping mechanism bypassing the CIP* intermediate. This is additionally supported by the ratio of the longer decay times 
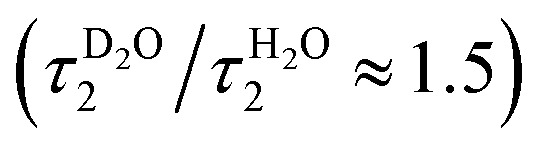
 that is equal to the ratio of the proton diffusion constants in H_2_O and D_2_O.^62^ Hence the slower deprotonation appears to proceed similarly to normal proton diffusion in aqueous environment. The overall biphasic decay, on the other hand, probably stems from a distribution of hydrogen-bonding environments that require a different extent of reorganization to facilitate the escape of the proton.

The decays in organic solvents exhibit more intricate behavior and were modeled using a three-exponential function. The multi-exponential behavior results in much poorer agreement between the experimental decays and the relative concentrations (Fig. 4A) that is particularly noticeable in DMSO. In DMSO, the ROH* population exhibits a bi-exponential initial decay with time constants *τ*_1_ = 3.9 ps and *τ*_2_ = 36 ps followed by an additional slower component. The fast initial decay is accompanied by a rise of the CIP* emission with comparable time constants (*τ*_1_ = 4.5 ps and *τ*_2_ = 23 ps). The first time constant is close to the mean solvation time, indicating that the initial step is partly driven by solvent relaxation. The second time constant is, however, significantly slower than the solvent relaxation and ESPT in this case must occur from the equilibrium solvent configuration. This suggests the presence of a solvent-induced reaction barrier^34,63^ although a large-scale solvent reorientation as a cause for the deceleration cannot be fully excluded. The last time constant (*τ*_3_ = 600 ps) can be attributed to the diffusion-controlled separation into free ions and it coincides with the rise time of the RO^−^* population (*τ* = 650 ps, Table S6†). It should be noted, that the reversibility of the initial step results in excited-state equilibrium between the ROH* and CIP* populations which is supported by the plateau in relative CIP* intensity reached after *ca.* 60 ps. Therefore, the RO^−^* rise time is not directly related to the inverse rate constant but depends additionally on the relative CIP* population.^35^

In alcohols, the time constants cannot be assigned to certain processes due to the competing ESPT pathways evidenced by the spectra of the deprotonated species. Similar to the aqueous environment, the different deprotonation pathways are likely facilitated by a distribution of local hydrogen-bonding environments. Some solvent configurations can facilitate the direct deprotonation *via* the hopping mechanism whereas a sub-population will deprotonate *via* the CIP* intermediate that is clearly discernible in MeOH. Besides the distribution of local solvent environments, reversibility of the second step can further complicate the kinetics and interpretation of the decay times is not straightforward.^64^ Therefore, the time constants are discussed only qualitatively.

In both alcohols, the shortest time constants are close to the mean solvation times showing partial involvement of the solvent relaxation in the reaction dynamics. In MeOH, the rise of the extracted CIP* emission around 20 × 10^3^ cm^−1^ follows a bi-exponential behavior with time constants *τ*_1_ = 3.2 ps and *τ*_1_ = 45 ps, again comparable to the first two decay constants of the ROH* population (5.1 ps and 55 ps). However, the RO^−^* emission also appears on the same time scale with a time constant of *τ* = 57 ps (Table S6†), similar to *τ*_2_ of the ROH* decay. In EtOH, the rise time (*τ* = 78 ps, Table S6†) is in between the first two ROH* decay components (*τ*_1_ = 29 ps and *τ*_2_ = 220 ps). In both alcohols, the longest decay component (*τ*_3_) of the ROH* form is significantly larger than the rise of the RO^−^* emission contrary to the situation in DMSO and aqueous solutions. This further supports the reversibility of the diffusion-controlled separation delaying the decay of the ROH* population.

## 3 Conclusions

Our results reveal several new insights into mechanistic aspects of ESPT to solvent. The dynamics in all solvents are multi-exponential suggesting the presence of a kinetic intermediate, namely the contact ion pairs (CIP*), in the reaction cycle. However, this intermediate is resolved spectrally only in MeOH and DMSO. In aqueous solvents (H_2_O and D_2_O), ESPT follows largely bi-exponential kinetics where the two time scales coincide with solvent relaxation and global hydrogen-bond rearrangement processes. This likely stems from a distribution of local hydrogen-bonding environments that require a different extent of reorganization to facilitate the escape of the proton. In both cases, the deprotonation produces free ions with largely red shifted fluorescence without spectrally distinguishable CIP* intermediate.

In alcohols, two competing pathways, a direct and *via* the CIP* intermediate, are clearly identified. Similarly to aqueous environment, this is attributed to a distribution of local hydrogen-bonding environments. Moreover, the diffusion-controlled separation becomes reversible as a result of the higher (more positive Δ*G*) reaction free energy in alcohols. In DMSO, the ESPT mechanism follows the Eigen–Weller model and proceeds in a step-wise manner. However, the initial decay of ROH* and formation of CIP* is bi-exponential and partially driven by the solvent relaxation. Full deprotonation into free ions occurs on a significantly longer time scale without any indications of reversibility.

General agreement between the experimental data and the global analysis based on the Eigen–Weller model is surprisingly good in aqueous solvents and satisfactory in alcohols and DMSO. However, the clear mechanistic differences in different solvents cast doubt on the validity of the rate constants obtained. The Eigen–Weller model is unable to account for multi-exponential dynamics that might arise from the influence of solvent relaxation or competing deprotonation pathways. Both of these effects are frequently observed in ultrafast ESPT to solvent limiting the applicability of the Eigen–Weller model. Therefore, the physical relevance of the rate constants obtained should be verified by other approaches and be in line with mechanistic considerations.

## Conflicts of interest

There are no conflicts of interest to declare.

## Supplementary Material

SC-011-D0SC03316B-s001
