# Supplementary material for: Broadband fluorescence reveals mechanistic differences in excited-state proton transfer to protic and aprotic solvents
Source: Chem Sci. 2020 Jul 8;11(30):7963–71. doi: 10.1039/d0sc03316b (PMC8163259; doi:10.1039/d0sc03316b)
Supplement: SC-011-D0SC03316B-s001 [file SC-011-D0SC03316B-s001.pdf]

Electronic Supplementary Material (ESI) for Chemical Science.
This journal is © The Royal Society of Chemistry 2020

Broadband Fluorescence Reveals Mechanistic Differences in Excited-State Proton Transfer to Protic and Aprotic Solvents

---

Electronic Supplementary Information

Pragya Verma,† Arnulf Rosspeintner,† Bogdan Dereka,†,‡ Eric Vauthey,† and Tatu Kumpulainen*,†

†*Department of Physical Chemistry, Sciences II, University of Geneva, 30, Quai Ernest Ansermet, 1211 Geneva, Switzerland*

‡*Current address: Department of Chemistry and Institute for Biophysical Dynamics, James Franck Institute, The University of Chicago, 929 E. 57th St., Chicago, IL 60637, USA*

E-mail: tatu.kumpulainen@unige.ch

Contents

S1 Experimental section **S3**

S1.1 Materials and methods S3

S1.2 Sample preparation and conditions S3

S2 Data analysis methods **S4**

S2.1 Determination of photophysical parameters S4

S2.2 Analysis of the time-resolved fluorescence spectra S5

S3 Spectral and photophysical parameters **S7**

S4 Time-resolved broadband fluorescence **S9**

S4.1 Experimental setup S9

S4.2 Solvation dynamics in H_2O and D_2O S10

S4.3 Time-resolved fluorescence upon excitation of the ROH form S11

S4.3.1 Fit details and applied boundary conditions S12

S4.3.2 Estimating the goodness of the fit S13

S4.3.3 Supplementary data and best-fit parameters S13

S4.4 Extracting the fluorescence spectra of the deprotonated species S18

S4.5 Multi-exponential analysis S20

S4.6 Comparing the global target analysis with the decay traces S20

S5 Femtosecond mid-IR transient absorption **S22**

S5.1 Experimental setup S22

S5.2 Time-resolved transient absorption upon excitation of the ROH form S23

S5.2.1 Supplementary data S23

References **S25**

S1 Experimental section

S1.1 Materials and methods

The 1,8-naphthalimide photoacid, SHONI, was synthesized according to the previously published procedure.[1] Briefly, 1,8-naphthalic anhydride was sulfonated with fuming sulfuric acid to give sodium-1,8-naphthalic anhydride-3,6-disulfonate.[2] The disulfonate was converted to potassium-3-hydroxy-1,8-naphthalic anhydride-6-sulfonate by reaction with molten KOH at 200–230 °C. Reaction with a primary amine, 2-(2-aminoethoxy)ethanol, yielded the target compound as pale yellow crystals. The compound was further recrystallized from acidified EtOH (first recrystallization) and acidified MeOH (second recrystallization).

Both quantum yield standards were of fluorescence grade (Coumarin 153 from Radiant dyes, Quinine sulfate from Sigma-Aldrich) and used as received. Dimethyl sulfoxide (DMSO, HPLC +99.9 % ChemSeal, Alfa Aesar), methanol (Uvasol, Sigma-Aldrich), absolute ethanol (EtOH, +99.5 %, Acros Organics), D_2O (for NMR, 99.8 % in D, Acros Organics), acetonitrile (MeCN, +99.9 %, Sigma-Aldrich), 1 N NaOH aq. (standard solution, Alfa Aesar) and 1 N HCl (standard solution, Fluka) were used as received. Demineralized water was used in all experiments.

Steady-state absorption spectra were recorded using a Varian Cary 50 spectrophotometer and emission spectra on a Horiba Scientific FluoroMax-4 fluorimeter, corrected using a set of secondary emissive standards.[3] The pH of aqueous solutions was monitored using a Mettler Toledo Seven2Go pH-meter calibrated with standard buffer solutions (Mettler Toledo). The ns time-correlated single photon counting has been described in detail previously.[4] The broadband femtosecond up-conversion setup is briefly described in Section S4.1.

S1.2 Sample preparation and conditions

Samples were prepared by dissolving a small amount of the target compound in either MeOH or in demineralized H_2O and diluting further to achieve an optimal absorption for each measurement. The stock solvent in MeOH was evaporated before addition of the organic solvent of interest. The pH (or protonation state) was controlled by adding small amounts of HCl aq. or NaOH aq. into the solvent of interest. For D_2O, deuterated acids and bases were used. The spectra of the protonated and the deprotonated forms were measured at ca. pH 3 and 12, respectively. In alcohols and DMSO, the same amount of acids and bases were used as in aqueous solutions.

The steady-state UV-vis absorption spectra were measured at relatively high absorption values ($A \approx 1$) whereas the emission spectra were measured with diluted samples ($A \leq 0.2$)

using a cuvette with 1 cm optical path length. The concentrations of the samples for the time-resolved measurements were adjusted based on the absorption at the excitation wavelength ($A = 0.2-0.4$ at 400 nm). All measurements were performed at room temperature (21±2 °C).

S2 Data analysis methods

S2.1 Determination of photophysical parameters

The fluorescence quantum yields were determined upon direct excitation of both the neutral form in acidic media and the anionic form in basic media. The quantum yields, Φ_f, were calculated according to:

$$\Phi_\mathrm{f,x} = \Phi_\mathrm{f,st} \cdot \frac{F_x}{F_\mathrm{st}} \cdot \frac{A_\mathrm{st} \cdot 10^{-A_\mathrm{st} \cdot l_\mathrm{p}}}{A_x \cdot 10^{-A_x \cdot l_\mathrm{p}}} \cdot \left(\frac{n_x}{n_\mathrm{st}}\right)^2, \quad \text{(S1)}$$

where F is the integrated fluorescence, A is absorption at the excitation wavelength, and n is the refractive index of the solvent. The subscripts 'x' and 'st' stand for the sample and standard, respectively. The exponential terms in the above equation account for the primary inner filter effect and the instrument specific l_p was determined to be 0.569 cm following the procedure from ref. 5. Moreover, the fluorescence spectra were corrected for the secondary inner filter effect (reabsorption) at each wavelength according to $F(\lambda)_\mathrm{corr} = F(\lambda)_\mathrm{obs} \cdot 10^{A(\lambda) \cdot l_\mathrm{p2}}$, with $l_\mathrm{p2} = 0.52$ cm.

The quantum yields were measured using two reference compounds, quinine sulfate dihydrate in 0.5 M H_2SO_4 (**QS**, $\Phi_\mathrm{f} = 0.55$)[6] and Coumarin 153 in EtOH (**C153**, $\Phi_\mathrm{f} = 0.53$),[7] as the quantum yield standards. The quantum yield of **QS** in 0.5 M H_2SO_4 was determined to be 56% using **C153** as the standard and this value was used for further calculations. After this cross referencing, both standards yielded nearly identical quantum yield values.

Absorption transition dipole moments were calculated according to:[8]

$$|M_\mathrm{abs}|^2 = \frac{3hc}{8\pi^3} \frac{\ln(10)}{N_\mathrm{A}} \frac{1}{f(n)} \frac{1}{n} \int_{abs} \frac{\epsilon(\tilde{\nu})}{\tilde{\nu}} d\tilde{\nu}, \quad \text{(S2)}$$

where h is Planck's constant, c the speed of light, N_A Avogadro's constant, n the solvent refractive index, and $\epsilon(\tilde{\nu})$ the molar absorption coefficient. The integration over $\epsilon(\tilde{\nu})$ was carried out from a low frequency side up to the local minimum in $\epsilon(\tilde{\nu})$ between $S_1 \leftarrow S_0$ and $S_2 \leftarrow S_0$ transitions located at around 28×10^3 cm^{-1} for ROH and 26.5×10^3 cm^{-1} for RO^- forms.

Emission transition dipole moments were calculated according to:[8]

$$|M_{\rm em}|^2 = \frac{3hc^3}{64\pi^4}\frac{1}{f(n)}\frac{1}{n^3}\frac{k_{\rm rad}}{\tilde{\nu}_f^3}, \tag{S3}$$

where $\tilde{\nu}_f^3 = \int F(\tilde{\nu})\tilde{\nu}^{-3}d\tilde{\nu}/\int F(\tilde{\nu})d\tilde{\nu}$ and $k_{\rm rad} = \Phi_{\rm fl}/\tau_{\rm fl}$.

In both above equations, the local-field correction factor, $f(n)$, proposed originally by Bakshiev was used.[9]

$$f(n) = 9n^2/(2n^2+1)^2 \tag{S4}$$

This corresponds to an empty spherical cavity and has been shown to produce consistent values for radiative rates and transition dipole moments in range of solvents of varying refractive indices.[8,10] Without this correction, the transition dipole moments in DMSO were significantly smaller due to the higher refractive index compared to the other solvents.

S2.2 Analysis of the time-resolved fluorescence spectra

The global analysis of the broadband fluorescence data for a single fluorescent species has been described in detail in ref. 11 and implementation of the target analysis including multiple fluorescent species in ref. 12. The main advantage of the analysis is that it enables independent evaluation of the solvent relaxation from the band positions and the population dynamics from the band integrals.

In the analysis, each fluorescent species is modeled with a time-dependent band shape function. The time-dependent band shape functions are constructed from log-normal functions according to eq. (S5),[13,14] in which the time-dependence of the band shape parameters is modeled using a constant or a multi-exponential function.

$$f(\tilde{\nu},t) = I(t)\begin{cases} \exp\left[-\ln(2)\left\{\ln\left(1+a(\tilde{\nu},t)\right)/b\right\}^2\right] & a(\tilde{\nu},t) > -1 \\ 0 & a(\tilde{\nu},t) \le -1 \end{cases} \tag{S5}$$

$$a(\tilde{\nu},t) = \frac{2b(\tilde{\nu}-\tilde{\nu}_0(t))}{\Delta x} \tag{S6}$$

In the above equations, $I(t)$ is the peak intensity, b the asymmetry parameter, $\tilde{\nu}_0(t)$ the peak frequency, and Δx the width parameter. The integral of the band, $A(t)$, can be calculated according to eq. (S7).

$$A(t) = I(t)\frac{\Delta x}{2}\exp\left[\frac{b^2}{4\ln 2}\right]\sqrt{\frac{\pi}{\ln 2}} \tag{S7}$$

In the present work, the global fit with the target model is achieved by using three time-dependent band shape functions, each corresponding to one of the excited-state species

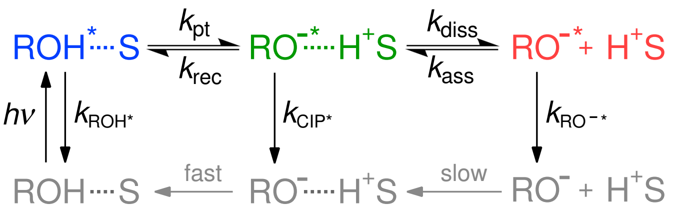

$$\frac{\mathrm{d}}{\mathrm{d}t}\begin{pmatrix}\mathbf{ROH^*}\\ \mathbf{CIP^*}\\ \mathbf{RO^{-*}}\end{pmatrix} = \begin{pmatrix}-(k_{\mathrm{pt}}+k_{\mathrm{ROH^*}}) & k_{\mathrm{rec}} & 0\\ k_{\mathrm{pt}} & -(k_{\mathrm{rec}}+k_{\mathrm{CIP^*}}+k_{\mathrm{diss}}) & k_{\mathrm{ass}}\\ 0 & k_{\mathrm{diss}} & -(k_{\mathrm{ass}}+k_{\mathrm{RO^{-*}}})\end{pmatrix}\cdot\begin{pmatrix}\mathbf{ROH^*}\\ \mathbf{CIP^*}\\ \mathbf{RO^{-*}}\end{pmatrix}$$

$$\text{with}\begin{pmatrix}\mathbf{ROH_0}\\ \mathbf{CIP_0}\\ \mathbf{RO_0^{-*}}\end{pmatrix} = \begin{pmatrix}1\\ 0\\ 0\end{pmatrix}$$

Scheme S1: (top) The reaction scheme used for target analysis of the broadband fluorescence spectra. The ground-state species indicated in gray are irrelevant for the fluorescence experiments. (bottom) Differential equations describing the concentrations of each excited-state species in the matrix notation. These equations are solved analytically to obtain the concentrations at each time step during the fitting procedure.

(ROH*, CIP* and RO⁻*). The fluorescence bands are allowed to undergo a dynamic Stokes shift due to the solvent relaxation, which is manifested as a frequency downshift of the peak maxima, $\tilde{\nu}_0(t)$. In this work, the solvent relaxation is modeled with a bi-exponential function with equal time constants for all species. The target model is taken into account by connecting the band integrals of the species, $A_i(t)$ in eq. (S7), through the differential equations presented in Scheme S1. The ground-state species indicated in gray are, of course, irrelevant for the fluorescence experiments. Since the data is analyzed in the transition dipole moment (TDM) representation, i.e. $F(\tilde{\nu})/\tilde{\nu}^3$,[15] the band integrals are directly proportional to the concentrations.[16] The differential equations are solved analytically using the matrix method described in ref. 17 to obtain the relative concentrations of the species at each time step. The concentration profiles are additionally convolved with a Gaussian-simulated instrument response function (IRF) to account for the finite duration of the excitation pulse. This enables fitting of the whole dataset before $t = 0$, which increases the accuracy of the parameter estimation at short times close to the IRF.

A synthetic dataset is generated as a sum of the three band shape functions according

to eq. (S8) and compared with the experimental data.

$$Y_{\text{fit}}(\tilde{\nu}, t) = \sum_{i=1}^{3} f_i(\tilde{\nu}, t) \tag{S8}$$

All fit parameters are simultaneously optimized through multiple iterations of the nonlinear least-square fitting routine on the minimization function according to eq. (S9).

$$f_{\text{min}} = \sum \left(\frac{Y_{\text{fit}}(t, \tilde{\nu}) - Y_{\text{data}}(t, \tilde{\nu})}{\text{SER}} \right)^2 \tag{S9}$$

where $Y_{\text{fit}}(t, \tilde{\nu})$ is the simulated and $Y_{\text{data}}(t, \tilde{\nu})$ the measured dataset, and SER is the standard error of the data. The SER is obtained from the standard deviations on each data point from multiple scans divided by the square root of the number of scans. All the above functions are modeled in matrix form, which significantly speeds up the optimization procedure. All analyses were performed using custom written Matlab scripts.

S3 Spectral and photophysical parameters

Spectral and photophysical properties of the protonated (ROH) and deprotonated (RO^-) forms were determined in slightly acidic or alkaline solutions, respectively. Frequencies of the absorption and emission band maxima were determined by fitting the experimental spectra in the TDM representation with a log-normal function around the band maxima. Absorption and emission band maxima are summarized in Table S1.

Fluorescence quantum yields were measured in duplicate using two different standards, both yielding nearly identical values. The fluorescence lifetimes were determined using a previously described time-correlated single photon counting setup with a time resolution of ca. 200 to 300 ps.[4] The lifetimes of the RO^{-*} forms correspond to the slower decay components of bi-exponential fits, where the faster decay component most likely originates

Table S1: Frequencies of the absorption and emission band maxima of SHONI in all solvents. The spectral parameters are given in wavenumber (in 10^3 cm^{-1}) and refer to values obtained in transition dipole moment representation

solvent	$\tilde{\nu}_{\text{abs}}^{\text{ROH}}$	$\tilde{\nu}_{\text{flu}}^{\text{ROH}}$	$\tilde{\nu}_{\text{abs}}^{\text{RO}^-}$	$\tilde{\nu}_{\text{flu}}^{\text{RO}^-}$
H_2O	26.0	∼22	22.5	14.5
D_2O	26.1	∼22	22.5	14.5
MeOH	26.0	22.2	22.6	14.8
EtOH	25.9	22.6	22.2	15.0
DMSO	25.7	22.3	19.5	15.4

Table S2: Photophysical parameters of SHONI in all solvents. The fluorescence quantum yields are given in %, lifetimes in ns and transition dipole moments in Debye

solvent	$\lvert M_{abs}^{ROH}\rvert$	$\lvert M_{abs}^{RO^-}\rvert$	$\Phi_{fl}^{tot\,a}$	τ_{fl}^{ROH}	$\lvert M_{fl}^{ROH}\rvert$	$\Phi_{fl}^{RO^-\,a}$	$\tau_{fl}^{RO^-}$	$\lvert M_{fl}^{RO^-}\rvert$
H_2O	2.7	2.8	0.6	–	–	0.7	0.5	2.7
D_2O	2.7	2.8	1.6	–	–	2.1	1.4	2.8
MeOH	2.7	2.8	1.5	–	–	2.8	1.6	3.0
EtOH	2.7	2.8	2.9	–	–	5.4	3.0	2.9
DMSO	2.7	2.9	24.0	–	–	26.1	15.4	2.7
MeCN	–	–	18.0	3.0	2.9	–	–	–

[a] The fluorescence quantum yield, Φ_{fl}^{tot}, corresponds to the total quantum yield ($ROH^* + RO^{-*}$) upon excitation of the ROH form whereas $\Phi_{fl}^{RO^-}$ corresponds to the fluorescence quantum yield of the RO^{-*} form upon direct excitation in alkaline solution.

from solvent relaxation and is too fast to be resolved with the current system. The lifetime of the ROH^* form in MeCN corresponds to the time constant of a mono-exponential fit.

The transition dipole moments (TDMs) of both the ROH and RO^- forms were determined from the absorption and emission according to eqs. (S2) and (S3), respectively. Due to the significant spectral overlap of the absorption bands corresponding to the $S_1 \leftarrow S_0$ and $S_2 \leftarrow S_0$ transitions in the ROH form, the band areas were estimated by decomposing the overall spectrum to a sum of two band shape functions. Displaced harmonic oscillator (DHO) model, eq. (S10),[18] was used as the band shape function to account for the noticeable vibronic structure.

$$\mathrm{DHO}(\nu) = \sum_{m=0}^{10} \frac{S^m e^{-S}}{m!} \exp\left\{\frac{-(h\nu_0 + m\hbar\omega - h\nu)^2}{2\sigma^2}\right\} \tag{S10}$$

In the above equation, S is the Huang-Rhys factor, ν_0 the 0–0 frequency, ω the harmonic frequency of the vibrational mode, and σ the width factor of individual vibronic bands. The above band shape function perfectly reproduces the experimental spectra as demonstrated in Figure S1. Since the two transitions are well separated in the spectra of the RO^- form, integration was carried out up to the local minima between the two transitions.

Emission TDMs of the RO^- form were determined in all solvents in slightly alkaline solutions. Since **SHONI** undergoes ESPT in protic solvents and DMSO, it was not possible to determine the emission TDM of the ROH form in these solvents. Therefore, neutral acetonitrile was used instead. **SHONI** does not undergo ESPT in acetonitrile most likely due to decreased proton solvating ability of acetonitrile[19] thus facilitating determination of both the intrinsic fluorescence quantum yield and lifetime of the ROH form. The quantum yields, lifetimes and TDMs are given in Table S2.

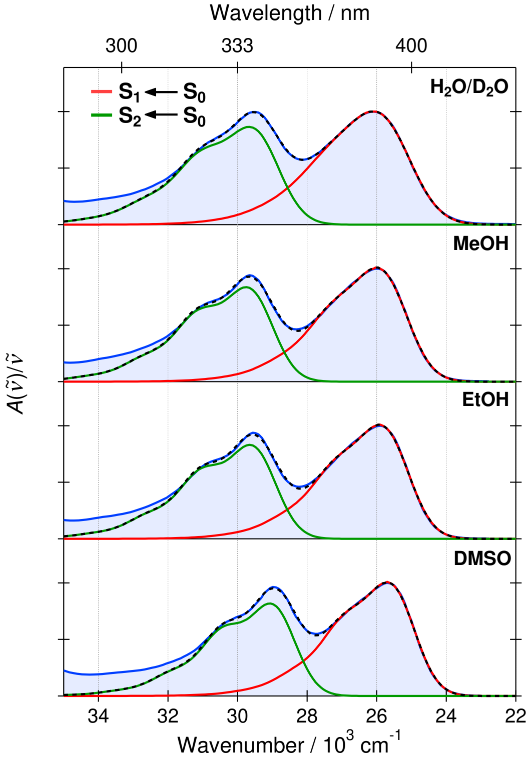

Figure S1: Absorption spectra (blue) of **SHONI** decomposed to into contributions from to the $S_1 \leftarrow S_0$ (red) and $S_2 \leftarrow S_0$ (green) transitions. The black dashed lines indicate the sum of the two band shape functions.

S4 Time-resolved broadband fluorescence

S4.1 Experimental setup

Broadband FLUPS measurements were preformed on a setup similar to that described in detail in refs. 20 and 21. In brief, excitation was performed with ~ 100 fs pulses at 400 nm generated by frequency doubling part of the output of a standard 1 kHz Ti:Sapphire amplified system. The pump intensity on the sample was below 1 mJ/cm^2. The gate pulses at 1340 nm were produced by an optical parametric amplifier (TOPAS Prime, Light Conversion). Detection of the up-converted spectra was performed with a home-built spectrograph coupled to a CCD camera (Andor, DV420A-BU). The full width at half-maximum (FWHM) of the cross correlation of the gate with the solvent Raman signal was approximately 170–180 fs. Time-resolved emission spectra were recorded in two sequential measurements with a linear time grid from –2 up to 4 ps and with a logarithmic time grid extending up to 1.5 ns depending on the sample. The measurement consisted of 5 to 10 successive scans with 2 s collection time at each time step. The raw data were transferred into spectra vs. wavenumber and subsequently corrected by calibration with secondary emissive standards as described

in ref. 20. Finally, the spectra were transferred into the TDM representation by diving with $\tilde{\nu}^3$.[15] The chirp polynomial due to group velocity dispersion was determined by measuring the instantaneous response of **BBOT** in the solvent of interest. The polynomial function was then used to remove the chirp from the experimental spectra of the photoacid.

S4.2 Solvation dynamics in H_2O and D_2O

As discussed in the main text, ESPT in H_2O and D_2O is extremely fast and proceeds on the same time scale as the solvent relaxation. As a result, the ROH* population decreases substantially during the first few ps compromising a detailed analysis of the solvation dynamics. Therefore, the time constants of the dynamic Stokes shift were determined independently upon direct excitation of the RO^{-*} form and used as constants in the analysis of the ESPT dynamics in H_2O and D_2O.

The time-resolved fluorescence of the deprotonated form was measured in H_2O and D_2O in alkaline solutions (NaOH aq.). The fluorescence spectra exhibit a dynamic Stokes shift and excited-state decay of the RO^{-*} form. The data was analyzed as described in Section S2 by assuming a single fluorescent species. The shifts were modeled using a bi-exponential function. The time-resolved fluorescence spectra, global fits and weighted residuals of the RO^{-*} form in H_2O and D_2O are presented in Figure S2. The best-fit parameters for the

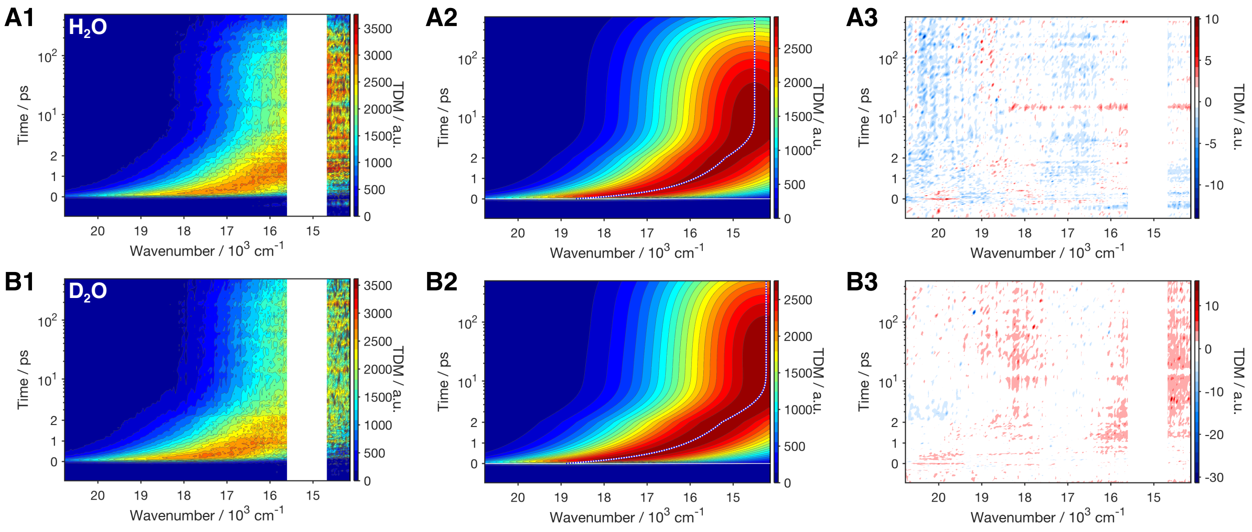

Figure S2: A) Time-resolved fluorescence of the RO^{-*} form of SHONI in H_2O (top) and D_2O (bottom); B) global fit ($\chi_r^2 = 1.67$ in H_2O; $\chi_r^2 = 2.34$ in D_2O) with a single fluorescent species; and C) weighted residual, all in the TDM representation and plotted on a lin-log time axis. The dashed white/blue line in B represents the time-dependent band maxima of the RO^{-*} form. The blank white areas are excluded from the fit due to the third harmonic of the gate pulse. Excitation was at 400 nm.

Table S3: The best-fit parameters for the dynamic Stokes shift and population decay of the RO^{-*} form in H_2O and D_2O. The spectral parametes are given in 10^3 cm^{-1}

solvent	$\Delta\tilde{\nu}_1$ (α_1)	τ_1	$\Delta\tilde{\nu}_2$ (α_2)	τ_2	$\tilde{\nu}_\infty$	$<\tau_{solv}>^a$	τ_{fl}	χ^2_r
H_2O	1.53 (0.36)	260 fs	2.69 (0.64)	1.5 ps	14.5	1.0 ps	0.5 ns	1.67
D_2O	1.61 (0.35)	320 fs	3.04 (0.65)	1.8 ps	14.2	1.3 ps	1.5 ns	2.34

[a] The mean solvation time was calculated according to $<\tau_{solv}> = \Sigma\alpha_i\tau_i$ where the amplitudes, α_i, are given in percentage.

dynamic Stokes shift and population decay are given in Table S3.

In both solvents, the RO^{-*} fluorescence band undergoes a large time-dependent red shift of >4000 cm^{-1} during the first few ps. The shifts exhibit an ultrafast component of ca. 300 fs and a longer component of 1.5–1.8 ps. The slower solvation component has an amplitude of 2690 and 3040 cm^{-1} in H_2O and D_2O, respectively, in close agreement with red shift of the RO^- absorption band in DMSO (~3000 cm^{-1}). The shift of the RO^- absorption band in DMSO is attributed to the lack of direct hydrogen bond between the deprotonated hydroxyl group and solvent. This suggests that the slower component is associated with the HB cleavage, requiring a significant reorientation of the neighboring water molecules. The mean solvation times (1.0 ps in H_2O and 1.3 ps in D_2O) are somewhat larger than some of the values reported in the literature.[22,23] This can be attributed to a much poorer time resolution of the instrument used in the present study (FWHM ≈ 170–180 fs). Therefore, the fastest sub-100 fs component is not resolved in our experiment. However, solvation times comparable to the values obtained here have been reported in some other studies with a comparable time resolution.[24] Secondly, deceleration of the solvent relaxation in deuterated water has been observed before.[25,26] Hence, the values reported here appear reasonable, considering the time resolution of our instrument. It should be additionally noted that the analysis of the spectral relaxation in the other studies was performed in the wavenumber representation contrary to the TDM representation used in the present study. Therefore, the values are not fully comparable from different studies. In any case, the resulting time constants for the dynamic Stokes shift determined here were constrained in the analysis of the ESPT dynamics in these two solvents.

S4.3 Time-resolved fluorescence upon excitation of the ROH form

The time-resolved fluorescence upon excitation of the protonated form was measured in H_2O, D_2O, MeOH, EtOH and DMSO in weakly acidic solutions. The absorption of the photoacid in neat solvents showed the presence of a small fraction of the deprotonated form, which was particularly noticeable in DMSO. The pH was decreased by adding few μL of 1 M HCl

aq. to ensure full protonation of the -OH group of the photoacid. The concentration of the photoacid was ca. 500 μM in all solvents. Excitation was at 400 nm in all cases.

S4.3.1 Fit details and applied boundary conditions

The spectra were analyzed globally using the Eigen-Weller model as described in Section S2. Certain assumptions were imposed in order to limit the number of free variables:

1. The TDMs of the different species were assumed to be identical
2. The solvent relaxation was assumed to be bi-exponential with equal lifetimes for all species
3. The widths (Δx) and asymmetries (b) of all species were assumed to remain constant
4. The relaxed RO^{-*} band position ($\tilde{\nu}_{\infty}$) was limited close ($\pm$200 cm^{-1}) to the value observed in the steady-state spectra
5. In organic solvents, the RO^{-*} band position ($\tilde{\nu}(t)$) was assumed to remain constant
6. The direct decay of the CIP* form (k_{CIP^*}) was assumed to be equal to that of the RO^{-*} form

Most of the assumptions are justified based on the experimental data. However, the direct decay of the CIP* form is difficult to access. Therefore, it was assumed to be equal to the decay of the RO^{-*} form whereas the decay of the ROH* form was used as an adjustable fit parameter. In effect, the direct decay of the ROH* form acts as the main loss channel in the process due to the rather long lifetime of the RO^{-*} form. However, the kinetic parameters for the ESPT processes were not significantly influenced by the choice of the loss channel i.e. whether it occurs via the ROH* or CIP* decay channel. The main difference was on the actual direct decay rate. The direct decay rate was significantly higher if the CIP* was selected as the main loss channel while fixing the k_{ROH^*} to the decay rate observed in MeCN (1/3000 ps^{-1}). This is likely due to the lower relative concentration of CIP* compared to ROH*. On the other hand, the direct decay rates of the RO^{-*} form resulting from the target analysis are somewhat different than the inverse lifetimes of the exponential decays. This disagreement might partially result from the constraint on the k_{CIP^*}.

Reversibility of the diffusion-controlled separation had to be taken into account in alcohols. The fit quality improved from $\chi_r^2 = 2.90$ to 2.16 in EtOH and from 2.66 to 2.39 in MeOH compared to the unidirectional separation. In MeOH, the reverse rate of the second step was significantly smaller ($(k_{\mathrm{ass}})^{-1} \approx 2.2$ ns) compared to that observed in EtOH ($(k_{\mathrm{ass}})^{-1} \approx 1.2$ ns). The other ESPT rate constants were largely impacted by the reversibility of the second step in EtOH whereas the impact was much smaller in MeOH as expected

based on the smaller reverse rate. In H_2O, D_2O, and DMSO, no improvement on the fit quality was observed upon inclusion of the reversibility suggesting irreversible separation.

Lastly, some constraints on the fit parameters were imposed. For example, in MeOH and DMSO an additional local minima was found where the forward and backward rates were significantly larger than the values presented in Table 1 of the main text. In these cases, the concentration profile of the ROH* did not match the observed decay because the spectral component of the CIP* accounted for a significant portion of the ROH* decay whereas the solvation dynamics were accounting for some of the population dynamics. Due to this reason, some constraints on the solvent relaxation parameters had to be imposed. Secondly, the time constants for the solvent relaxation in H_2O and D_2O were constrained to values determined upon direct excitation of the RO^{-*} form (see Table S3). In these solvents, the ESPT reaction is extremely fast and occurs during the solvent relaxation. In any case, we tried to use as few constraints as possible and all the rate parameters for the ESPT process were freely adjustable. All constraints (upper/lower boundary or fixed) are clearly indicated in Table S4 and S5 together with the best-fit parameters.

S4.3.2 Estimating the goodness of the fit

Goodness of the fit was judged by looking at the weighted residuals as well as the reduced χ^2 value. In all cases, satisfactory fits with χ_r^2 ranging from 2.0 to 3.1 was achieved. Nevertheless, significant patterns was observed in the residuals in all cases. This is partly due to limitations of the log-normal function to adequately reproduce the spectral band shapes and partly due to inadequacies of the target model. In MeOH and EtOH, the competing dissociation pathways are not accurately captured by the target analysis based on the Eigen-Weller model, hence giving rise to the patterns in the residuals (see main text for details). On the other hand, in DMSO the target analysis is unable to reproduce the multi-exponential formation of the CIP* population. Due to these reasons, the χ_r^2 values are much higher and residuals show more deviations in alcohols and DMSO.

S4.3.3 Supplementary data and best-fit parameters

The time-resolved fluorescence spectra, global fits and weighted residuals in all solvents are presented in Figure S3 as 3D-surface plots and in Figure S4 as 2D-spectra. The concentration profiles all species and exponential fittings of the ROH* and RO^{-*} forms (*vide infra*) in all solvents are presented in Figure S5. The best-fit parameters for the band shape functions of all species and the solvation dynamics of the ROH* form are summarized in Tables S4 and S5, respectively. The kinetic parameters and χ_r^2 values are given in Table 1 of the main text.

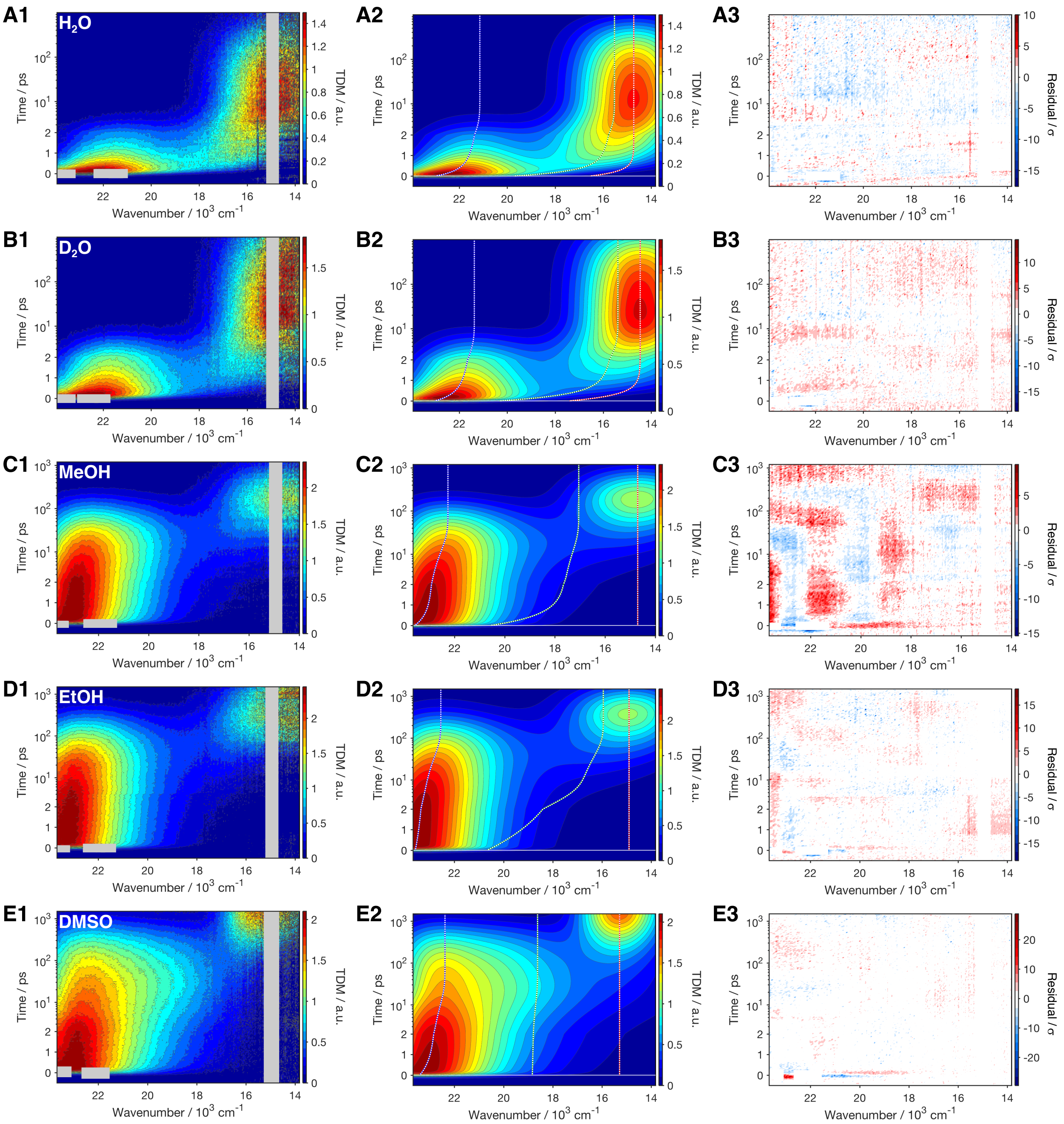

Figure S3: 1) Experimental time-resolved fluorescence spectra, 2) global fits and 3) weighted residuals of SHONI in A) H_2O, B) D_2O, C) MeOH, D) EtOH and E) DMSO as 3D-surface plots. The spectra and fits are in TDM representation whereas the weighted residuals are given in standard deviations, all plotted on a lin-log time axis. The dashed lines in 2) represents the time-dependent band maxima of each species. The gray areas are excluded from the fit due to the residual pump, raman scattering and third harmonic of the gate pulse. Excitation was at 400 nm.

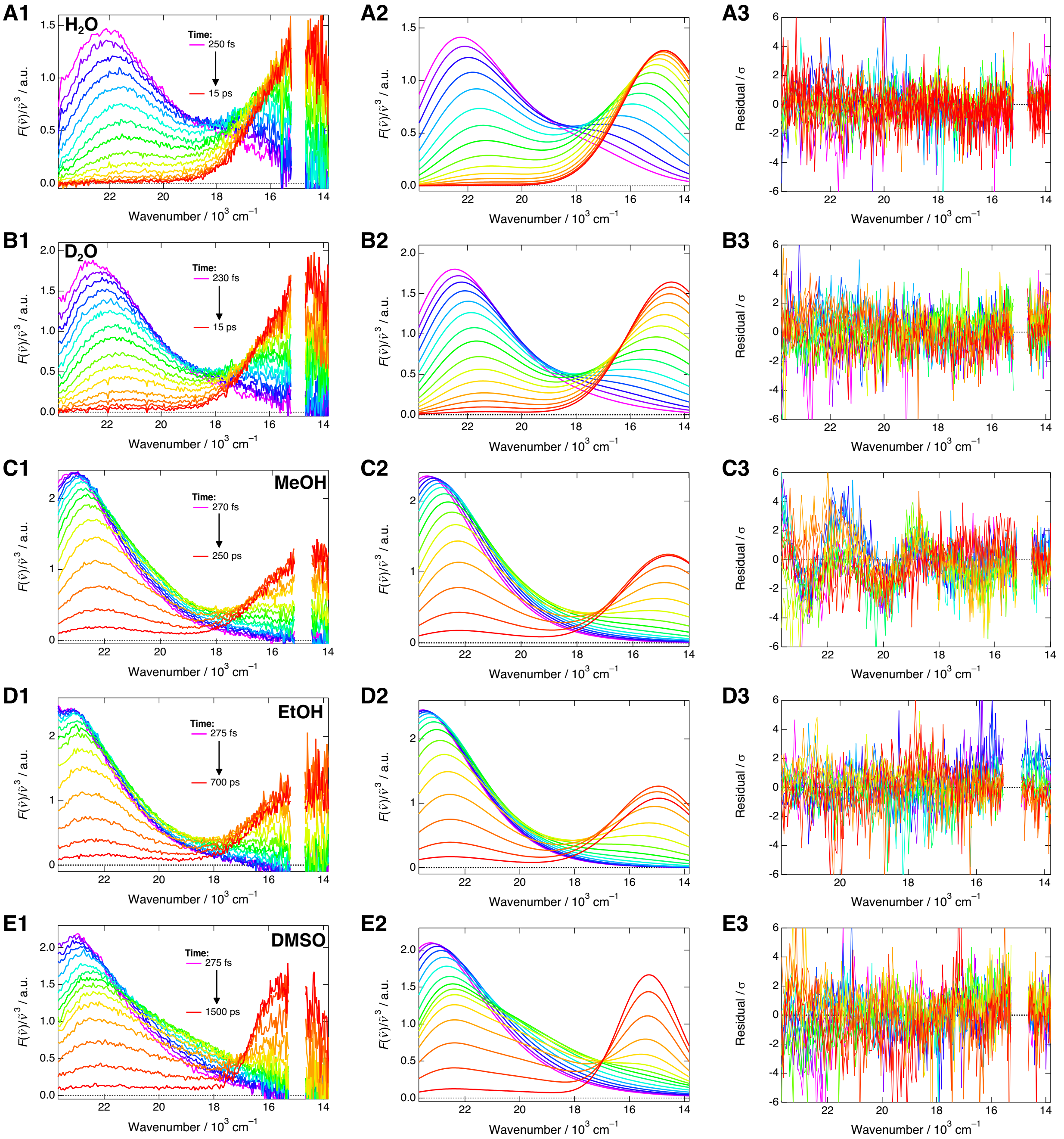

Figure S4: 1) Experimental time-resolved fluorescence spectra, 2) global fits and 3) weighted residuals of SHONI in A) H_2O, B) D_2O, C) MeOH, D) EtOH and E) DMSO at selected time steps as 2D-spectra. The time steps are spaced on a logarithmic grid between the values indicated in the legends. The spectra and fits are in TDM representation whereas the weighted residuals are given in standard deviations. The blank white areas are excluded from the fit due to the third harmonic of the gate pulse. Excitation was at 400 nm.

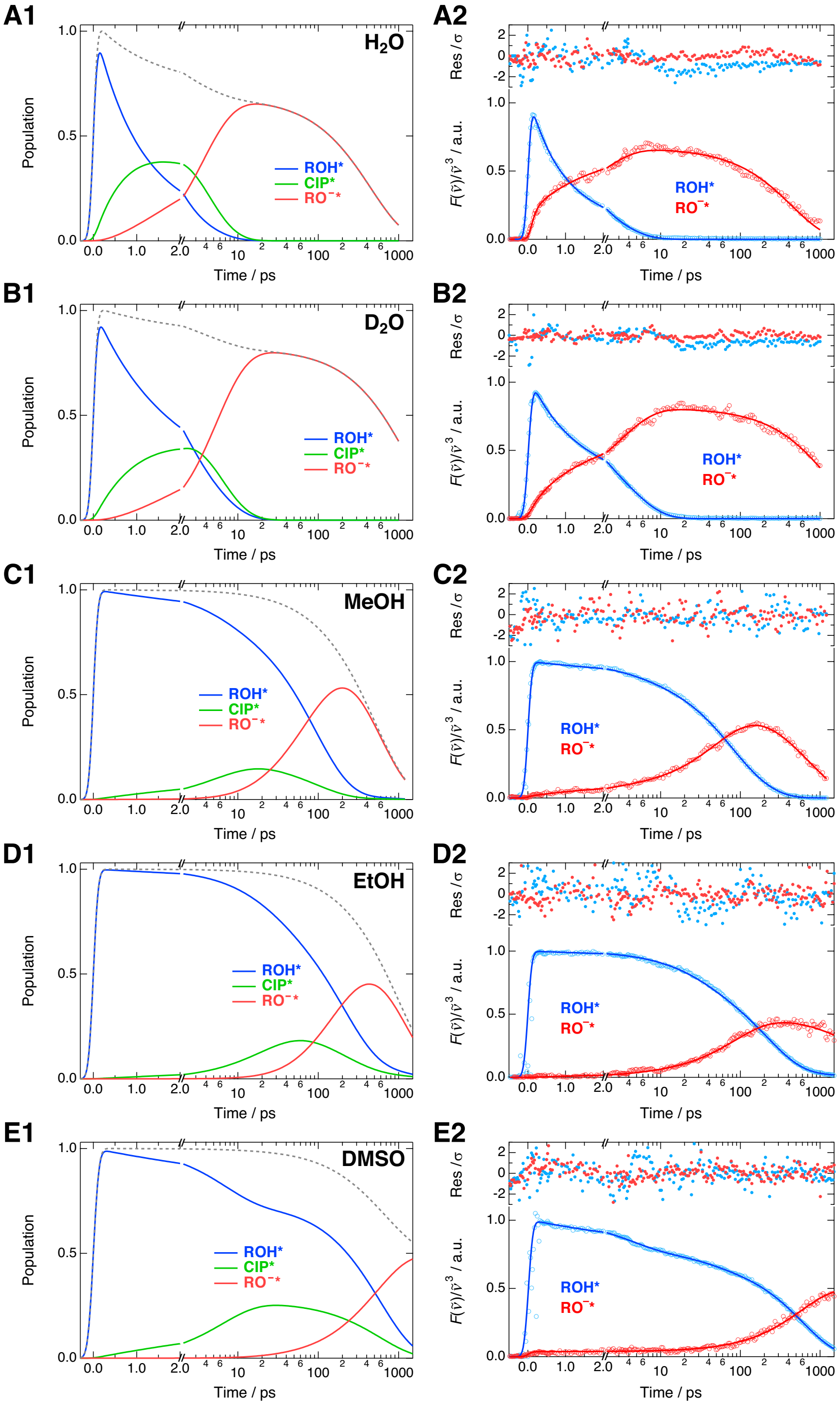

Figure S5: 1) Concentration profiles of all species resulting from the global target analysis. The corresponding rate constant are given in Table 1 of the main text. The dashed gray lines indicate the total excited-state population. 2) Time traces and exponential fits of the ROH* and RO^{-*} forms after compensating for the spectral shifts of the ROH* form (*vide infra*). The exponential fit parameters and spectral ranges are given in Table S6. The weighted residuals are given in the top panels. All profiles are plotted on a lin-log time axis. The solvents are A) H_2O, B) D_2O, C) MeOH, D) EtOH and E) DMSO.

Table S4: Summary of the band shape parameters of all species obtained from the global target analysis of the time-resolved fluorescence spectra. All spectral parameters (except b) are given in 10^3 cm^{-1}

	ROH*					CIP*					RO^{-*}				
solvent	$\Delta\tilde{\nu}_1$	$\Delta\tilde{\nu}_2$	$\tilde{\nu}_\infty$	Δx	b	$\Delta\tilde{\nu}_1$	$\Delta\tilde{\nu}_2$	$\tilde{\nu}_\infty$	Δx	b	$\Delta\tilde{\nu}_1$	$\Delta\tilde{\nu}_2$	$\tilde{\nu}_\infty$	Δx	b
H_2O	1.1	0.9	21.1	4.1	-0.3	3.6	1.0	15.5	4.1	0.0^a	2.0^a	0.0	14.7	3.6	0.0^a
D_2O	0.8	0.8	21.4	3.9	-0.3	4.0	1.0	15.4	4.7	-0.1	3.0^a	0.0	14.5	3.8	0.0
MeOH	0.6	0.8	22.3	3.6	-0.4	3.0^a	0.6	17.0	5.9	0.0^a	–	–	14.7^b	3.9	-0.1
EtOH	0.3	0.7	22.6	3.7	-0.3	3.6	1.1	15.9	5.1	0.0^a	–	–	14.9^b	3.5	0.0^a
DMSO	0.5	0.5	22.4	3.9	-0.5	0.0	0.2	18.3	5.2	-0.6	–	–	15.3^b	2.6	0.0

^{a} The upper limit of the parameter was constrained to this value; ^{b} The lower limit of the parameter was constrained to this value.

Table S5: Summary of the solvent relaxation parameters of the ROH* form obtained from the global target analysis of the time-resolved fluorescence spectra. All spectral parameters are given in 10^3 cm^{-1}

solvent	α_1	τ_1/ps	α_2	τ_2/ps	$\Delta\tilde{\nu}_{tot}$	$\tilde{\nu}_\infty$	$\tilde{\nu}_{SS}$	$<\tau_{solv}>$/ps
H_2O	0.55	0.25^a	0.45	1.5^a	1.95	21.1	~22	0.8±0.1
D_2O	0.50	0.31^a	0.50	1.8^a	1.66	21.4	~22	1.1±0.1
MeOH	0.43	0.54	0.57	11.3	1.35	22.3	22.2	6.7±0.3
EtOH	0.30	2.2	0.70	34.0^b	1.04	22.6	22.6	25±1
DMSO	0.50	0.76	0.50	9.7^b	1.05	22.4	22.3	5.2±0.3

^{a} The parameter was constrained to this value; ^{b} The upper limit of the parameter was constrained to this value.

Table S6: Relative amplitudes and lifetimes of the ROH* and RO^{-*} forms obtained from the exponential fittings after compensating for the spectral shift of the ROH* form ^{a}

	ROH*						RO^{-*} ^{b}				
solvent	α_1	τ_1/ps	α_2	τ_2/ps	α_3	τ_3/ps	α_1	τ_1/ps	α_2	τ_2/ps	τ_3/ns
H_2O	0.60	0.7	0.40	2.5	–	–	-0.35	0.1	-0.65	1.75	0.4
D_2O	0.37	0.8	0.63	3.9	–	–	-0.19	0.4	-0.81	3.1	1.3
MeOH	0.09	5.1	0.42	55	0.49	130	-0.08	1.8	-0.92	57	0.8
EtOH	0.20	29	0.75	220	0.05	1500	–	–	-1.0	78	4.9
DMSO	0.21	3.9	0.10	36	0.69	600	–	–	-1.0	650	15.0

^{a} The time traces in Figure S5 correspond to average intensities close to the band maxima after compensating for the spectral shift of the ROH* form (see Figure S6 in section S4.4). The intensities were averaged over 20.5–21.0 and 15.3–15.9 in H_2O and D_2O, 22.0–22.1 and 15.4–15.6 in MeOH, 22.3–22.5 and 15.4–15.6 in EtOH, and 22.4–22.6 and 15.4–15.6 in DMSO, for the ROH* and RO^{-*} forms, respectively. The ranges are given in 10^3 cm^{-1}; ^{b} The amplitudes α_1 and α_2 for the RO^{-*} form correspond to the rise whereas τ_3 corresponds to the excited-state decay with an amplitude of one.

S4.4 Extracting the fluorescence spectra of the deprotonated species

The neat fluorescence spectra of the deprotonated species (CIP^* and RO^{-*}) were extracted using the approach described in our earlier publication.[12] Briefly, the spectral shift of the ROH^* form was removed from the data by shifting the spectra at each time step to match the peak frequencies to the value of the fully relaxed ROH^* fluorescence. Time-dependent peak frequencies of the ROH^* form were taken from the results of the global analysis presented above. The shifting effectively removes all contributions from the solvent relaxation to the ROH^* fluorescence but does not necessarily compensate for the shifts of the CIP^* and RO^{-*} fluorescence. The spectra were subsequently normalized at the ROH^* peak maxima and the first spectrum was subtracted from the subsequent spectra. This removes all fluorescence contributions originating from the ROH^* form. The first spectrum, used for the subtraction, was taken at around 250–300 fs after disappearance of the signals due to pump and Raman scattering processes. In H_2O and D_2O, the reaction is extremely fast and a small contribution from the deprotonated species is subtracted as well. Nevertheless, the kinetics still correspond to the true rise but the amplitude of the rise component is slightly reduced depending on the amount of the deprotonated fluorescence in the first spectrum. The procedure is demonstrated in Figure S6 in all solvents. The left panels show the original spectra, the middle panels the shifted and normalized spectra and the right panels the final subtracted spectra corresponding to the fluorescence due to the deprotonated species.

In MeOH and DMSO, a clearly distinguishable CIP^* fluorescence band is observed after the subtraction procedure. In both cases, the CIP^* spectrum is relatively broad and peaks at an intermediate frequency between the ROH^* and RO^{-*} fluorescence bands. The peak frequency of the CIP^* band appears to remain rather stationary in both solvents. This shows that the dynamic Stokes shift (i.e. solvation free energy) of the CIP^* form is of the same magnitude as that of the ROH^* band. Band maxima of the CIP^* form is closer to the ROH^* form in MeOH ($\sim$20.0 $\times 10^3$ cm^{-1}) whereas it is shifted more towards the RO^{-*} form in DMSO ($\sim$18.5 $\times 10^3$ cm^{-1}). Such a shift is indicative of increased driving force of the forward reaction resulting from significantly stronger stabilization of the excited-state product. Similar conclusion have been reported in a computational study on contact ESPT reactions.[27] The driving force has been additionally shown to influence the bond lengths along the proton-transfer coordinate. The hydrogen-bond length between the deprotonated photoacid and the protonated solvent increases upon increasing driving force.[27] Hence the spectral position of the CIP^* band additionally provides insight into the structure of the contact ion pairs. In DMSO, the proton is further away from the hydroxyl oxygen compared to MeOH.

The increased driving force in DMSO is also supported by the much higher intensity and

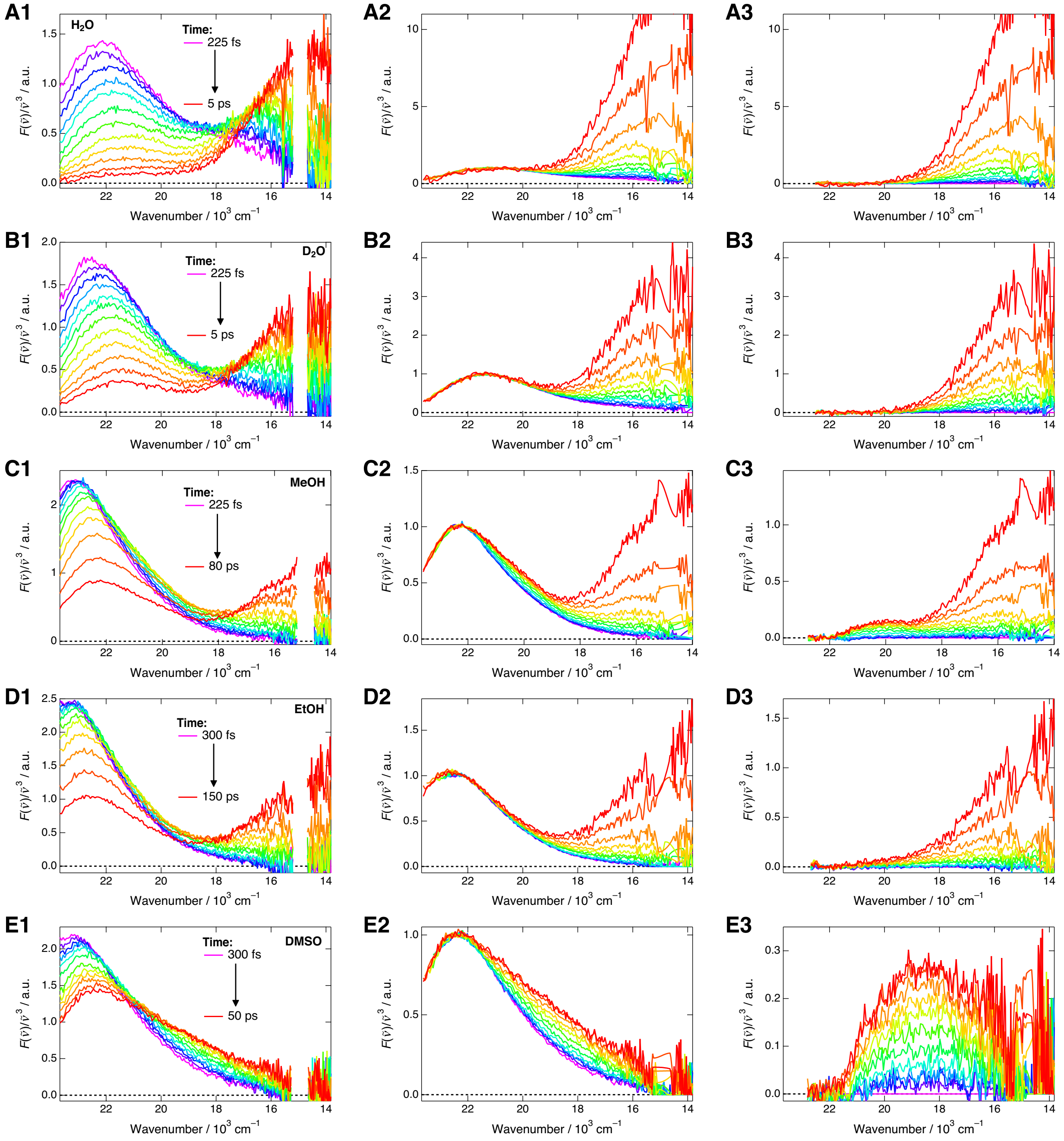

Figure S6: 1) Experimental time-resolved fluorescence spectra, 2) normalized spectra after removing the spectral shift of ROH* form, and 3) final subtracted spectra corresponding to the neat fluorescence due to the deprotonated species. The time steps are spaced on a logarithmic grid between the values indicated in the legends. The blank white areas are excluded spectra due to the third harmonic of the gate pulse. The wiggles around this region in the shifted spectra are artefacts from the shifting procedure.

hence the population of the CIP* form. In MeOH, the intensity of the CIP* band reaches 0.15 relative to the ROH* band whereas the intensity is nearly double (0.28) in DMSO. The

increased driving force in DMSO likely originates from a higher proton accepting ability of the solvent. This supported by the more negative proton solvation free energy in DMSO compared to that in MeOH reported in several computational studies.[19,28,29] In addition, pK_a of protonated DMSO is significantly higher (lower acidity) than that of protonated MeOH making neutral DMSO a stronger base.[30]

S4.5 Multi-exponential analysis

Multi-exponential analysis of the decay traces is usually challenging because the single wavelength traces contain contributions from both the spectral shifts as well as the population dynamics. Therefore, we extracted the decay traces from the spectra after removing the dynamic Stokes shift of the ROH* form. This additionally removes contributions from the solvent relaxation on the CIP* as discussed above. Hence the decay traces extracted around the ROH* and CIP* band maxima are not contaminated by the spectral shifts. However, the RO^{-*} form is expected to experience stronger solvent stabilization and hence the decay traces around the RO^{-*} band maxima might contain some contribution from the solvent relaxation. Because the excited-state decay is significantly slower than the solvent relaxation, the overall slow decay is free from artifacts and reports on the population dynamics.

The decay traces for the ROH* and RO^{-*} forms were obtained as an average intensity close to the corresponding band maxima. The exact spectral ranges used for averaging are given in the footnote of Table S6. The traces were analyzed with either two- or three-exponential functions convolved with a Gaussian instrument response function. The decay traces together with the exponential fits are presented in the right column of Figure S5. The resulting lifetimes and amplitudes are given in Table S6.

Due to the significant overlap with the ROH* band, the rise kinetics of the CIP* form were extracted from the shifted and normalized spectra after subtraction of the ROH* fluorescence. Therefore, the amplitude of the CIP* is given relative to the ROH* form and the multi-exponential rise reflects the time scale for the formation of the excited-state equilibrium between the ROH* and CIP* forms. The rise kinetics were analyzed with a two-exponential function. The rise traces in MeOH and DMSO together with the exponential fits are presented in Figure 4B of the main text.

S4.6 Comparing the global target analysis with the decay traces

The weighted residuals presented in the right panels of Figures S4 and S3 provide a general comparison between the experimental spectra and the global fit. However, visual comparison of the individual population dynamics with the experimental decay traces is not straightfor-

ward from these residuals. This owes to both the spectral overlap of different species as well as to the spectral shifts due to solvent relaxation. Therefore, we present here a graphical comparison between the relative populations of the ROH* form, $c_{\mathrm{rel}}(\mathrm{ROH}^*)$, obtained from the global target analysis and the corresponding decay traces extracted from the shifted spectra (*vide supra*). Both of these traces are free from signals due to spectral shifts and reflect purely the population dynamics of the ROH* from. The decay traces of the ROH* fluorescence are overlaid with the population profiles in Figure S7.

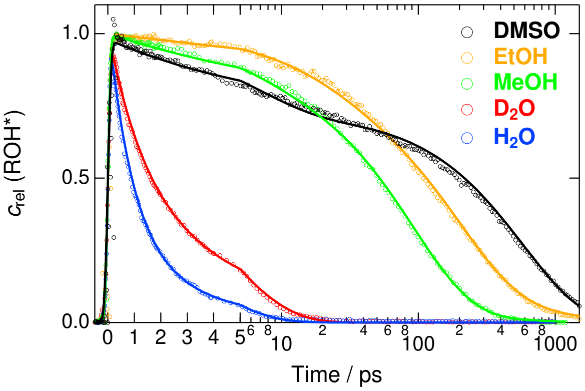

Figure S7: Decay traces of the ROH* fluorescence extracted from the shifted spectra (markers) overlaid with the relative populations of the ROH* form obtained from the global target analysis (lines).

The agreement between $c_{\mathrm{rel}}(\mathrm{ROH}^*)$ and the decay traces in H_2O and D_2O is excellent. This is also reflected by the rather small χ_r^2 values ($\sim$2) resulting from the global target analysis. The reason for the excellent agreement is the bi-exponential decay of the ROH* fluorescence in these two solvents (see Table S6). Analytical solution of the reaction kinetics according to the Eigen-Weller model in the case of an irreversible second step will also yield bi-exponential decay of the ROH* population.[31] The first time constant corresponds to the formation of the excited-state equilibrium between ROH* and CIP* whereas the second time constant corresponds to the decay of the equilibrium state and formation of the free ions. In this case, the exponential decay of the ROH* form is correctly reproduced by the Eigen-Weller model.

The agreement is much worse in organic solvents. The χ_r^2 value is highest in DMSO ($>$3) indicating the worst agreement between the fit and the experimental data. The poor fit quality can be reasoned based on the exponential analysis. The initial decay of the ROH* fluorescence and formation of CIP* is clearly bi-exponential. As discussed in the main text, the first time constant is close to the mean solvation time whereas the second time constant is significantly longer. This indicates that the part of the reaction occurs during

the solvent relaxation whereas the slower part suggest a significant reaction barrier in the equilibrium solvent configuration. After the bi-exponential formation, the equilibrium state decays on a longer time scale with a concomitant formation of the free ions. In DMSO, the Eigen-Weller model fails to account for this bi-exponential initial decay. This is also seen in Figure S7 as significant deviations between $c_{\text{rel}}(\text{ROH}^*)$ (black line) and the experimental decay (black markers). The fastest initial part of the experimental decay is missed in global target analysis. The same partly applies to MeOH although in both alcohols, the competing deprotonation pathways additionally complicate the behavior. As discussed in the main text, the competing pathways are likely resulting from a distribution of local hydrogen-bonding (HB) environments that require a different extent reorganization to facilitate ESPT. In protic solvents, the proton transfer can be highly sensitive to the local HB environment due to Grotthuss-type proton hopping mechanism.[32,33]

S5 Femtosecond mid-IR transient absorption

S5.1 Experimental setup

Broadband mid-IRTA spectra were obtained using a setup which has been described in detail elsewhere.[34] The setup is based on a Ti:Sapphire amplified system (Spectra Physics Solstice) producing 100 fs pulses at 800 nm at 1 kHz. Excitation was performed with ca. 1 μJ pulses at 400 nm produced by frequency doubling a fraction of the amplifier output. The intensity and the polarization was controlled with a zero-order half-wave plate followed by a Glan-Laser polarizer, limiting the time resolution of the experiment to 300 fs. The pulses were focused on the sample to a spot of 350 μm resulting in an irradiance of $\sim$0.3 mJ/cm^2. Mid-IR probe pulses at around 5.6–7.0 μm (aromatic and C=O stretch) where generated by difference frequency mixing of the output of an optical parametric amplifier (Light Conversion, TOPAS-C with NDFG module) that was pumped at 800 nm. The polarization of the IR beam was controlled using a wire-grid polarizer. Two horizontally polarized IR beams were produced with a CaF_2 wedge and focused onto the sample to a 140 μm diameter spot. One of the beams was overlapped with the pump beam, whereas the second one was used as a reference. Both IR beams were focused onto the entrance slit of an imaging spectrograph (Horiba, Triax 190, 150 lines/mm) equipped with a liquid nitrogen cooled 2×64 element MCT array (Infrared Systems Development), giving a resolution of 1–2 cm^{-1} throughout the detection window.

The sample area and the detection system were placed in a box that was purged with water-free and carbon dioxide-free air for at least one hour before and during each experiment. The average of 2000 signal shots was taken to collect one data point with the polarization

of the pump at the magic angle to that of the IR pulse. This procedure was carried out for at least ten times. To provide a new sample solution for each shot, a flow cell as described in ref. 35 was used. The absorbance at 400 nm was below 0.4 on a 100 μm optical path length used for DMSO. Thinner spacers (25–50 μm) and saturated solutions were used in protic solvents resulting in maximum absorbance below 0.1 at 400 nm. No significant sample degradation was observed throughout the experiments. The transient spectra recorded in three, ca. 100–150 cm^{-1}, spectral windows were first averaged and then merged to obtain a single continuous spectrum covering the full 1500–1800 cm^{-1} spectral range. No tail matching or scaling of the different regions had to be carried out.

S5.2 Time-resolved transient absorption upon excitation of the ROH form

The time-resolved UV-pump mid-IR-probe transient absorption spectra upon excitation of the protonated form were measured in D_2O, MeOH, and DMSO in weakly acidic solutions. Similarly to the fluorescence measurements, pH of the sample solutions was adjusted by adding few μL of 1 M HCl aq. to ensure full protonation of the -OH group of the photoacid. Due to the thinner sample cells (25–100 μm), concentration of the photoacid was significantly higher (several mM) corresponding to saturated solutions in protic solvents. In H_2O and EtOH, absorption of the photoacid in the saturated solutions was too weak compared to the solvent absorption to achieve reasonable signal-to-noise. In MeOH, only satisfactory signal-to-noise could be achieved. Excitation was at 400 nm in all cases.

S5.2.1 Supplementary data

The time-resolved mid-IR transient absorption spectra in D_2O, MeOH and DMSO are presented in Figure S8. The two main bleach signals originate from symmetric and anti-symmetric CO stretch vibrations located at around 1700 and 1660 cm^{-1} in all solvents. In D_2O, an additional bleach due to an aromatic stretch vibration of the 1,8-naphthalimide skeleton is visible at 1620 cm^{-1} whereas it overlaps with the induced absorption in MeOH and DMSO. There are two weaker aromatic stretch vibrations around 1600 cm^{-1} that are not clearly visible due to the induced absorption. All these bands were assigned in our previous publication based on the FTIR spectra and DFT calculations.[12]

The induced absorption bands are rather narrow and well defined in DMSO. Moreover, the signal-to-noise is much better in DMSO due to the higher solubility and lower solvent absorption. We demonstrated in our previous work that the same target model based on the Eigen-Weller scheme can be used to analyze the transient absorption spectra. Full analysis

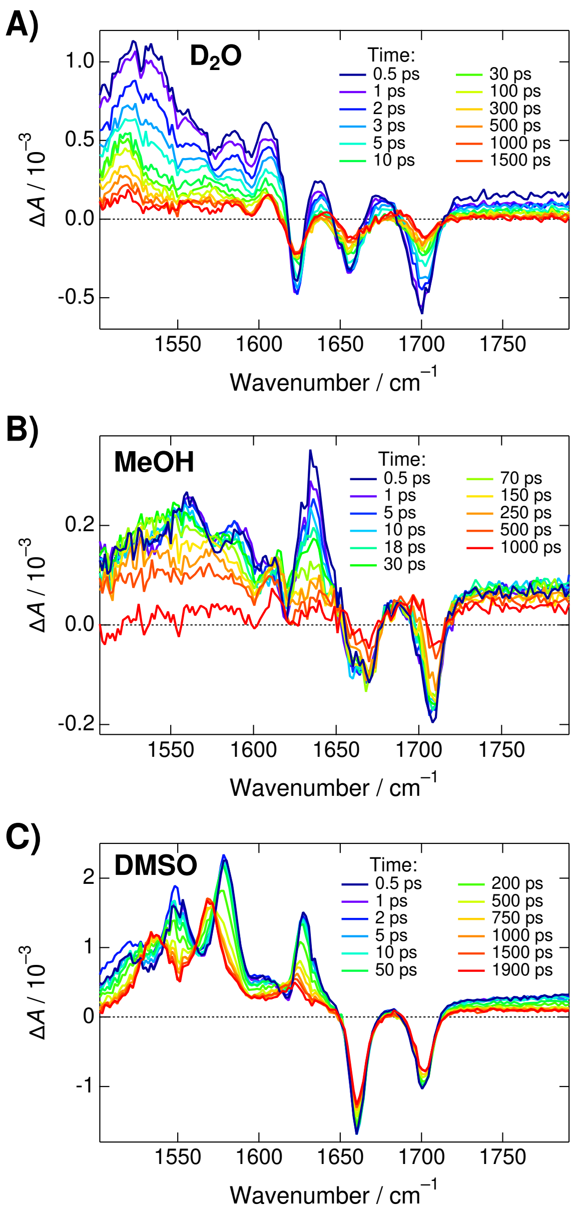

Figure S8: Time-resolved UV-pump mid-IR-probe transient absorption spectra of **SHONI** upon excitation of the ROH form in slightly acidic A) D2O, B) MeOH, and C) DMSO. Delay times of each spectra are indicated in the legends. Excitation was at 400 nm in all cases.

and interpretation of the spectra in DMSO is presented in ref. 12. We made an attempt to analyze the spectra in protic solvents but this did not results in physically meaningful species associated difference spectra. Simple global analysis is able to recover some of the time constants that are also observed in the exponential analysis but the signals due to solvation dynamics cannot be separated from the population dynamics. In both cases, the carbonyl and aromatic stretch vibrations undergo gradual red shifts. As discussed in the main text, both the solvation and population dynamics occur largely on the same time scale further hin-

dering the separation. Moreover, most of the induced absorption bands are extremely broad and featureless in protic solvents presumably due to different HB environments. Therefore, meaningful discussion on the spectral shifts is not feasible in protic solvents.

However, we want to highlight the strong continuum signal observed above 1650 cm^{-1} in all solvents. The signal is particularly strong in D_2O and MeOH. As discussed in the main text, similar continuum signals have been observed for other photoacids in protic solvents.[36,37] In one study,[37] the continuum signal was attributed to a strong and highly polarizable hydrogen bond. This represents the excited photoacid-solvent complex where the proton is shared by the two reaction partners. Therefore, the coupling between the solute and solvent modes is expected to be strong, allowing for an efficient dissipation of the excitation energy into the environment. Solvated protons in H_2O and D_2O have also very broad absorption bands in the mid-IR region[38–40] but their absorption cross-section has been suggested to be significantly smaller.[37] Our data supports this interpretation since the continuum signal appears directly upon excitation before the ESPT reaction. Moreover, the continuum signal decays significantly upon deprotonation especially in D_2O and DMSO. This indicates a significant reduction in the absorption cross section upon cleavage of the direct hydrogen bond. In MeOH, significant fraction of the continuum signal persists during the whole excited-state lifetime (>1 ns). Therefore, it is likely that significant part of the continuum signal in MeOH originates from solvated protons. This is supported by experimental IR-spectrum of solvated protons in MeOH and MeOH clusters although the absorption cross section relative to that in aqueous solutions has not been elucidated.[41] In any case, more detailed study into the nature of the continuum signals is beyond the present study.

References

(1) Kumpulainen, T.; Bakker, B. H.; Hilbers, M.; Brouwer, A. M. Synthesis and Spectroscopic Characterization of 1,8-Naphthalimide Derived “Super” Photoacids. *J. Phys. Chem. B* **2015**, *119*, 2515–2524.

(2) Dziewoński, K.; Majewicz, T.; Schimmer, L. Weitere Studien über die Naphthalsäure-disubstitutionsderivate. *Bull. Int. Acad. Pol. Sci. Lett., Cl. Sci. Math. Nat.,Ser. A* **1936**, 43–55.

(3) Gardecki, J. A.; Maroncelli, M. Set of Secondary Emission Standards for Calibration of the Spectral Responsivity in Emission Spectroscopy. *Appl. Spectrosc.* **1998**, *52*, 1179–1189.

(4) Muller, P.-A.; Högemann, C.; Allonas, X.; Jacques, P.; Vauthey, E. Deuterium isotope effect on the charge recombination dynamics of contact ion pairs formed by electron-transfer quenching in acetonitrile. *Chem. Phys. Lett.* **2000**, *326*, 321–327.

(5) Kubista, M.; Sjöback, R.; Eriksson, S.; Albinsson, B. Experimental Correction for the Inner-filter Effect in Fluorescence Spectra. *Analyst* **1994**, *119*, 417–419.

(6) Brouwer, A. M. Standards for Photoluminescence Quantum Yield Measurements in Solution (IUPAC Technical Report). *Pure Appl. Chem.* **2011**, *83*, 2213–2228.

(7) Würth, C.; Grabolle, M.; Pauli, J.; Spieles, M.; Resch-Genger, U. Relative and Absolute Determination of Fluorescence Quantum Yields of Transparent Samples. *Nat. Protoc.* **2013**, *8*, 1535–1550.

(8) Lewis, J. E.; Maroncelli, M. On the (Uninteresting) Dependence of the Absorption and Emission Transition Moments of Coumarin 153 on Solvent. *Chem. Phys. Lett.* **1998**, *282*, 197–203.

(9) Bakshiev, N.; Girin, O.; Libov, V. Relation Between the Observed and True Absorption Spectra of Molecules in a Condensed Medium III. Determination of the Influence of an Effective (Internal) Field According to the Models of Lorentz and Onsager-Böttcher. *Opt. Spectrosc. (U.S.S.R.)* **1963**, *14*, 395–398.

(10) Toptygin, D. Effects of the Solvent Refractive Index and its Dispersion on the Radiative Decay Rate and Extinction Coefficient of a Fluorescent Solute. *J. Fluoresc.* **2003**, *13*, 201–219.

(11) Kumpulainen, T.; Rosspeintner, A.; Vauthey, E. Probe Dependence on Polar Solvation Dynamics from fs Broadband Fluorescence. *Phys. Chem. Chem. Phys.* **2017**, *19*, 8815–8825.

(12) Kumpulainen, T.; Rosspeintner, A.; Dereka, B.; Vauthey, E. Influence of Solvent Relaxation on Ultrafast Excited-State Proton Transfer to Solvent. *J. Phys. Chem. Lett.* **2017**, *8*, 4516–4521.

(13) Rusch, P. F.; Lelieur, J. P. Analytical Moments of Skewed Gaussian Distribution Functions. *Anal. Chem.* **1973**, *45*, 1541–1543.

(14) Fraser, R. D. B.; Suzuki, E. Resolution of Overlapping Bands: Functions for Simulating Band Shapes. *Anal. Chem.* **1969**, *41*, 37–39.

(15) Angulo, G.; Grampp, G.; Rosspeintner, A. Recalling the Appropriate Representation of Electronic Spectra. *Spectrochim. Acta, Part A* **2006**, *65*, 727–731.

(16) Rumble, C. A.; Breffke, J.; Maroncelli, M. Solvation Dynamics and Proton Transfer in Diethylaminohydroxyflavone. *J. Phys. Chem. B* **2017**, *121*, 630–637.

(17) Berberan-Santos, M. N.; Martinho, J. M. G. The Integration of Kinetic Rate Equations by Matrix Methods. *J. Chem. Educ.* **1990**, *67*, 375.

(18) Arzhantsev, S.; Zachariasse, K. A.; Maroncelli, M. Photophysics of Trans-4-(dimethylamino)-4'-cyanostilbene and Its Use as a Solvation Probe. *J. Phys. Chem. A* **2006**, *110*, 3454–3470.

(19) Kelly, C. P.; Cramer, C. J.; Truhlar, D. G. Single-Ion Solvation Free Energies and the Normal Hydrogen Electrode Potential in Methanol, Acetonitrile, and Dimethyl Sulfoxide. *J. Phys. Chem. B* **2007**, *111*, 408–422.

(20) Zhang, X.-X.; Würth, C.; Zhao, L.; Resch-Genger, U.; Ernsting, N. P.; Sajadi, M. Femtosecond Broadband Fluorescence Upconversion Spectroscopy: Improved Setup and Photometric Correction. *Rev. Sci. Instrum.* **2011**, *82*, 063108.

(21) Gerecke, M.; Bierhance, G.; Gutmann, M.; Ernsting, N. P.; Rosspeintner, A. Femtosecond Broadband Fluorescence Upconversion Spectroscopy: Spectral Coverage Versus Efficiency. *Rev. Sci. Instrum.* **2016**, *87*, 053115.

(22) Pérez-Lustres, J. L.; Rodríguez-Prieto, F.; Mosquera, M.; Senyushkina, T. A.; Ernsting, N. P.; Kovalenko, S. A. Ultrafast Proton Transfer to Solvent: Molecularity and Intermediates from Solvation- and Diffusion-Controlled Regimes. *J. Am. Chem. Soc.* **2007**, *129*, 5408–5418.

(23) Sajadi, M.; Weinberger, M.; Wagenknecht, H.-A.; Ernsting, N. P. Polar Solvation Dynamics in Water and Methanol: Search for Molecularity. *Phys. Chem. Chem. Phys.* **2011**, *13*, 17768–17774.

(24) Spry, D. B.; Goun, A.; Fayer, M. D. Deprotonation Dynamics and Stokes Shift of Pyranine (HPTS). *J. Phys. Chem. A* **2007**, *111*, 230–237.

(25) Barbara, P. F.; Walker, G. C.; Kang, T.-J.; Jarzeba, W. Ultrafast Experiments on Electron Transfer. *Proc. SPIE* **1990**, *1209*, 18–31.

(26) Pant, D.; Levinger, N. E. Polar Solvation Dynamics of H2O and D2O at the Surface of Zirconia Nanoparticles. *J. Phys. Chem. B* **1999**, *103*, 7846–7852.

(27) Gamiz-Hernandez, A. P.; Magomedov, A.; Hummer, G.; Kaila, V. R. I. Linear Energy Relationships in Ground State Proton Transfer and Excited State Proton-Coupled Electron Transfer. *J. Phys. Chem. B* **2015**, *119*, 2611–2619.

(28) Farrokhpour, H.; Manassir, M. Approach for Predicting the Standard Free Energy Solvation of H+ and Acidity Constant in Nonaqueous Organic Solvents. *J. Chem. Eng. Data* **2014**, *59*, 3555–3564.

(29) Rossini, E.; Knapp, E. W. Proton Solvation in Protic and Aprotic Solvents. *J. Comput. Chem.* **2016**, *37*, 1082–1091.

(30) WadaGoro, Basicity of Dimethyl Sulfoxide in an Aqueous Solution as Measured by the Spectrophotometric Indicator Method. *Bull. Chem. Soc. Jpn.* **2006**, *42*, 890–893.

(31) Veiga Gutiérrez, M.; Brenlla, A.; Carreira Blanco, C.; Fernández, B.; Kovalenko, S. A.; Rodríguez-Prieto, F.; Mosquera, M.; Pérez-Lustres, J. L. Dissociation of a Strong Acid in Neat Solvents: Diffusion is Observed After Reversible Proton Ejection Inside the Solvent Shell. *J. Phys. Chem. B* **2013**, *117*, 14065–14078.

(32) Agmon, N. The Grotthuss Mechanism. *Chem. Phys. Lett.* **1995**, *244*, 456–462.

(33) Agmon, N. Elementary Steps in Excited-State Proton Transfer. *J. Phys. Chem. A* **2005**, *109*, 13–35.

(34) Koch, M.; Letrun, R.; Vauthey, E. Exciplex Formation in Bimolecular Photoinduced Electron-Transfer Investigated by Ultrafast Time-Resolved Infrared Spectroscopy. *J. Am. Chem. Soc.* **2014**, *136*, 4066–4074.

(35) Bredenbeck, J.; Hamm, P. Versatile Small Volume Closed-Cycle Flow Cell System for Transient Spectroscopy at High Repetition Rates. *Rev. Sci. Instrum.* **2003**, *74*, 3188–3189.

(36) Mohammed, O. F.; Dreyer, J.; Magnes, B.-Z.; Pines, E.; Nibbering, E. T. J. Solvent-Dependent Photoacidity State of Pyranine Monitored by Transient Mid-Infrared Spectroscopy. *ChemPhysChem* **2005**, *6*, 625–636.

(37) Siwick, B. J.; Bakker, H. J. On the Role of Water in Intermolecular Proton-Transfer Reactions. *J. Am. Chem. Soc.* **2007**, *129*, 13412–13420.

(38) Kampschulte-Scheuing, I.; Zundel, G. Tunnel Effect, Infrared Continuum, and Solvate Structure in Aqueous and Anhydrous Acid Solutions. *J. Phys. Chem.* **1970**, *74*, 2363–2368.

(39) Biswas, R.; Carpenter, W.; Fournier, J. A.; Voth, G. A.; Tokmakoff, A. IR Spectral Assignments for the Hydrated Excess Proton in Liquid Water. *J. Chem. Phys.* **2017**, *146*, 154507.

(40) Fournier, J. A.; Carpenter, W. B.; Lewis, N. H. C.; Tokmakoff, A. Broadband 2D IR Spectroscopy Reveals Dominant Asymmetric H5O2+ Proton Hydration Structures in Acid Solutions. *Nat. Chem.* **2018**, *10*, 932–937.

(41) Stoyanov, E. S.; Stoyanova, I. V.; Reed, C. A. IR Spectroscopic Properties of H(MeOH)n+ Clusters in the Liquid Phase: Evidence for a Proton Wire. *Chem. Eur. J.* **2008**, *14*, 3596–3604.
